# An Analysis of Nucleotide–Amyloid Interactions Reveals Selective Binding to Codon-Sized RNA

**DOI:** 10.1021/jacs.3c06287

**Published:** 2023-10-02

**Authors:** Saroj K. Rout, Riccardo Cadalbert, Nina Schröder, Julia Wang, Johannes Zehnder, Olivia Gampp, Thomas Wiegand, Peter Güntert, David Klingler, Christoph Kreutz, Anna Knörlein, Jonathan Hall, Jason Greenwald, Roland Riek

**Affiliations:** † Institute of Molecular Physical Science, ETH Zürich, 8093 Zürich, Switzerland; ‡ Institute of Technical and Macromolecular Chemistry, RWTH Aachen University, 52074 Aachen, Germany; § Max Planck Institute for Chemical Energy Conversion, 45470 Mülheim/Ruhr, Germany; ∥ Institute of Biophysical Chemistry, Goethe University, 60438 Frankfurt am Main, Germany; ⊥ Department of Chemistry, Tokyo Metropolitan University, Hachioji 192-0397, Japan; # Institute of Organic Chemistry and Center for Molecular Biosciences Innsbruck (CMBI), Universität Innsbruck, 6020 Innsbruck, Austria; 7 Institute of Pharmaceutical Sciences, ETH Zürich, 8093 Zürich, Switzerland

## Abstract

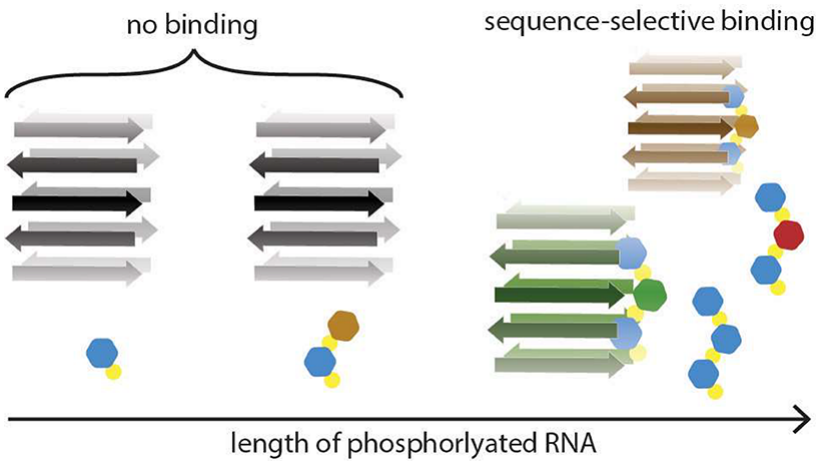

Interactions between RNA and proteins are the cornerstone of many important biological processes from transcription and translation to gene regulation, yet little is known about the ancient origin of said interactions. We hypothesized that peptide amyloids played a role in the origin of life and that their repetitive structure lends itself to building interfaces with other polymers through avidity. Here, we report that short RNA with a minimum length of three nucleotides binds in a sequence-dependent manner to peptide amyloids. The 3′–5′ linked RNA backbone appears to be well-suited to support these interactions, with the phosphodiester backbone and nucleobases both contributing to the affinity. Sequence-specific RNA–peptide interactions of the kind identified here may provide a path to understanding one of the great mysteries rooted in the origin of life: the origin of the genetic code.

## Introduction

**Figure 1:**
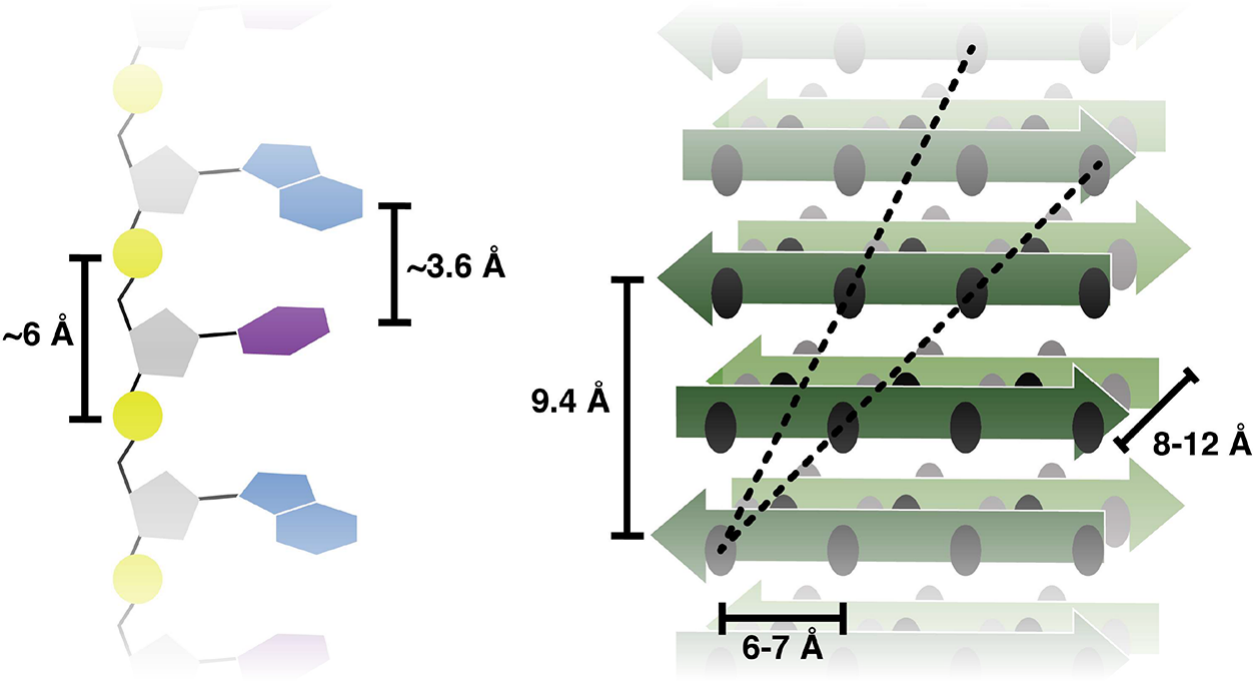
The periodic nature of RNA and amyloid structures. A schematic representation of RNA, on the left, is composed of phosphodiester linkages (yellow), ribose (gray), and nucleobases (purple and blue). The amyloid, on the right, is composed of β-structured peptides (green) that form β-sheets (here indicated as antiparallel although parallel is also possible) with their side chains (black) pointing alternately into and out of the fibril composed of two β-sheets. The repetitive distances that can occur in both RNA and amyloid structures are indicated. Note that parallel β-sheets have a repeating distance of 4.7 Å instead of 9.4 Å. The dashed lines on the β-sheets indicate other repetitive features on the amyloid surface.

Questions concerning the origin of life are often couched in terms of what sort of molecule arose first. The linear thinking in this approach to prebiotic chemistry, perhaps guided by a need to solve the chicken–egg paradox embedded firmly in the central dogma of molecular biology, is predestined to fall short of its goal. That is, the elaborate chemical networks that support life could not have originated from a few exceedingly complex molecules, but rather it is more likely that systems of simpler, more abundant molecules were involved. It has thus been hypothesized that prebiological polymers of different classes, in particular nucleic acids and peptides, co-evolved and thereby developed synergies that made them interdependent.^1−8^ Peptide amyloids have been shown to be prebiotically relevant entities with replicative and catalytic potential,^9−16^ and their structurally repetitive nature (Figure 1) provides a scaffold upon which nucleotide and fatty acid bilayer interactions can be stabilized.^17^ The multivalency of such binding partners allows for potentially high affinities to be reached through avidity-enhanced interactions. Due to their polyanionic nature, interactions with nucleic acids can be driven in large part by electrostatic complementarity with their phosphate groups.^18−23^ Amyloid–RNA interactions have been shown to accelerate RNA hydrolysis^24^ as well as protect RNA from alkaline hydrolysis,^25^ and cationic variants of an Aβ_16–22_ peptide have been shown to co-assemble with RNA oligonucleotides of a minimum length of six nucleotides to form ribbon-like structures.^18^ Furthermore, DNA oligonucleotides of a length of 33 or more nucleotides have been shown to induce amyloid formation of basic peptides, and conversely, amyloids can stabilize the hybridization of double-stranded DNA.^23^ In a recent study, the interaction between 20-nucleotide-long RNA duplexes and non-amyloid 11–16-mer cationic depsipeptides has been shown to significantly increase the thermal stability of the folded RNA structures as well as increase the resistance of the depsipeptides to hydrolytic degradation more than 30-fold.^19^ Taking into account the importance of electrostatics, we set out to explore the chemical and structural requirements that govern the interactions between small peptide amyloids and short RNA, including the RNA length and the role of the nucleobases in sequence-selective RNA binding.

## Results and Discussion

**Figure 2:**
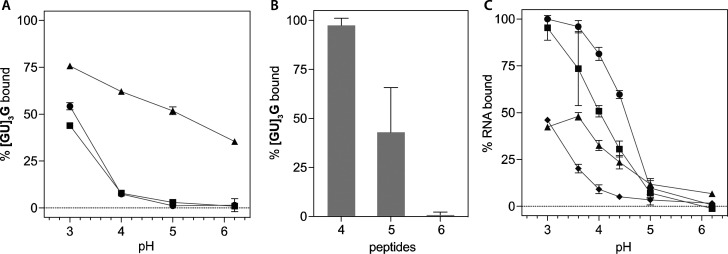
pH, charge, and sequence dependence of RNA–amyloid interactions. (A) The pH dependence of the interaction between [GU]_3_G and the amyloids of peptides **1**, **2**, and **3** (circles, squares, and triangles, respectively). (B) The interaction between [GU]_3_G and the amyloids of peptides **4**, **5**, and **6** at pH 3 demonstrates the requirement of a positive charge character on the amyloid. (C) Binding of peptide **7** to [GA]_3_G (circles), [GU]_3_G (squares), [CA]_3_C (triangles), and [CU]_3_C (diamonds) demonstrates the RNA sequence dependence of the interactions. The assays were performed at room temperature in a citrate–phosphate buffer with 100 μM peptide and 50 μM RNA oligos. Errors are given as the standard deviation of two completely independent assays.

Considering the known interactions between RNA and amyloids and their ability to stabilize each other, we investigated the molecular determinants of RNA–amyloid interactions. For this, we took advantage of the insolubility of amyloids in order to detect the binding of RNA, whereby mixtures of RNA and amyloids were centrifuged, and the supernatant RNA was quantitated by reverse-phase HPLC. RNA-only controls were used to quantitate the total RNA and to account for possible nonspecific binding of the RNA to the plastic DNA LoBind tubes (Eppendorf). An amyloidogenic nature for the peptides used in this study (Table 1) was expected based on the alternating hydrophilic/hydrophobic character of the sequences; however, we also measured the Fourier-transform infrared (FTIR) spectra of the aggregated samples to verify that they possess β-sheet structure, used transmission electron microscopy (TEM) to image their fibrillar nature, and determined the percentage of aggregation by HPLC (Figures S1–S3). Considering the importance of electrostatics in peptide–RNA interactions, we first investigated the pH dependence of the binding between the RNA heptanucleotide GUGUGUG ([GU]_3_G) and a series of peptide amyloids of varying electrostatic character. For these assays we used a stoichiometric excess of the peptide, with 100 μM peptide and 50 μM RNA. As shown in Figure 2A, peptide amyloids comprised of both acidic and basic ionizable groups have a strong pH dependence for their interaction with RNA. Low pH is more favorable for binding, which we attribute to the protonation and neutralization of carboxylate groups, thereby facilitating the interaction with the negatively charged phosphate backbone of RNA. Hence, the C-terminally amidated peptide **3** shows the lowest dependence on pH because it retains an overall positive charge character even at a neutral pH, while for peptides **2** and **1**, the C-terminus and the Glu side chain, unless protonated, can interfere with binding to the [GU]_3_G oligonucleotide. To further investigate the role of electrostatics, [GU]_3_G binding to peptide amyloids that contain multiple ionizable groups (peptides **4**, **5**, and **6**), including peptide **6** which has no cationic group due to the acetylation of its N-terminus, was measured at pH 3. The results show that more positive charges on the peptide yield more RNA binding and that at least a single positive charge, such as the N-terminus in peptide **5**, is required for binding (Figure 2B and Figure S4). The role of the RNA nucleobases in the binding process was analyzed with a series of RNA heptanucleotides: [GA]_3_G, [GU]_3_G, [CA]_3_C, and [CU]_3_C. For this analysis, we chose the highly ionizable peptide **7** which, relative to peptide **4**, has an additional N-terminal Val residue (the identity of which was also varied in a series of peptides designed to test specificity on the amyloid side) (**S4** of Table S1). The pH dependence of the binding of the RNA heptanucleotides to peptide **7** was similar to that observed with the binding to peptides **1** and **2** but also revealed a significant RNA sequence dependence (Figure 2C). For illustration purposes, the raw HPLC traces used to obtain the data in Figure 2C are depicted in Figure S5. Guanosine- and adenosine-containing sequences bind better than those with cytidine and uridine. While this sequence dependence is likely to be at least partially due to the varying hydrophobic and electrostatic nature of each nucleobase, it cannot be wholly explained by them. The order of hydrophobicity of the nucleotides, based on both the mononucleotide logP_octanol–water_ values^26^ and the retention times of the GNG trinucleotides on reverse-phase chromatography (Figure S6), is A > U > G > C. Based on their measured p*K*_a_ values, the positive charge character of the bases at acidic pH values should be C > A > G > U.^26^ One obvious trend is that sequences with purine bases bind better than those with pyrimidines.

**Table 1:**
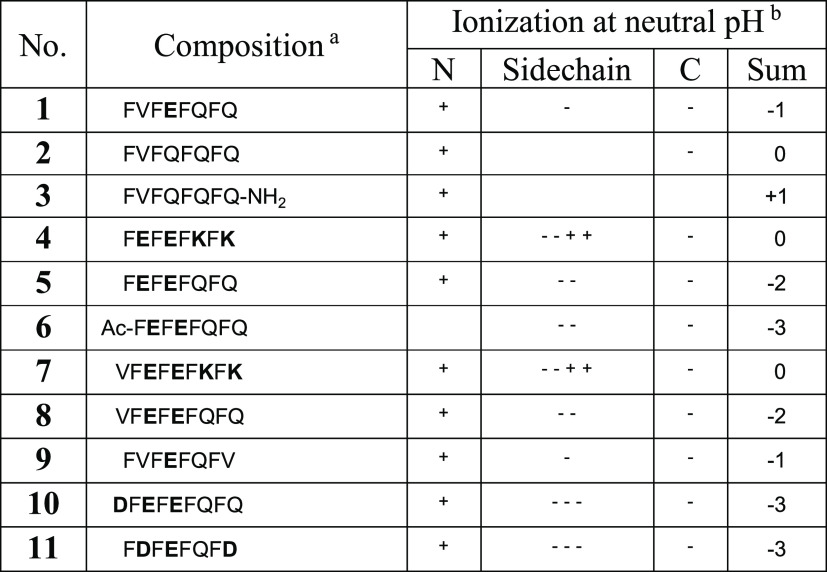
Amyloidogenic Peptides Used in This Study

a Amino acid residues are represented in the standard single letter code; Ac– is for an acetylated N-terminus and −NH_2_ is for an amidated C-terminus.

b Dominant ionization states of the ionizable groups of the peptide at neutral pH are listed as the charges on the N-terminus, side chains, and C-terminus. The listed ionization states are based on the individual p*K*_a_ values of the groups and are expected to vary depending on buffer pH and the aggregation state of the peptide.

**Figure 3:**
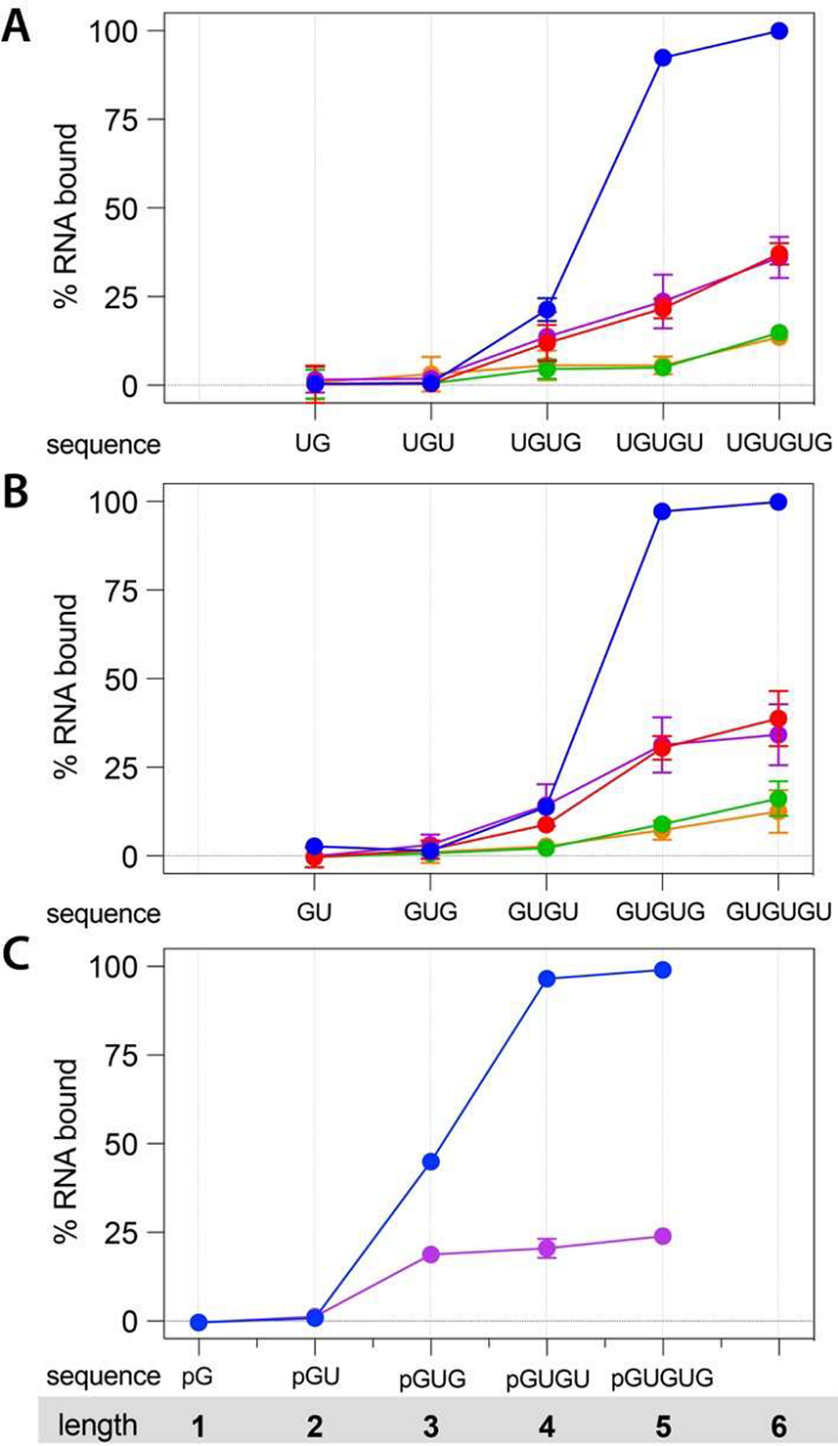
Oligonucleotide length dependence of RNA–amyloid interactions. Length-dependent binding of RNA to amyloids of different composition and charge character. (A) The binding of RNA sequences starting with U and alternating with G and (B) starting with G and alternating with U to the amyloids of peptides **7** (blue), **8** (red), **9** (purple), **10** (green), and **11** (orange). (C) Similar as in B but with 5′-phosphorylated RNA binding to peptides **7** (blue) and **9** (purple). The assays were performed at room temperature and pH 3 with 100 μM peptide and 25 μM RNA. Errors are given as the standard deviation of two completely independent assays.

Considering the nearly 100% binding of 50 μM [GU]_3_G to peptide **4** (Figure 2B), we reasoned that the apparently high affinity is a result of the avidity inherent in the interaction between two structurally repetitive entities. We therefore investigated the impact of RNA length dependence on the binding to peptide amyloids, using mononucleotides up to hexaribonucleotides each containing an alternating G/U sequence motif. As expected, due to avidity, there is a strong length dependence on the binding interaction (Figure 3). Interestingly, despite a large range in the overall binding of these RNAs to a range of different peptide amyloids, there appeared to be a consistent minimum length of four nucleotides at which interactions could be observed. Considering that a significant share of the affinity may come from the charge complementarity between the phosphodiester backbone and the positive charges on the amyloid, we reasoned that the four-nucleotide minimum length could be principally a three-phosphate minimum length. To test this, we measured the interactions of 5′-phosphorylated mono-, di-, and trinucleotides. The importance of the phosphate backbone in the interaction was confirmed with the 5′-phosphorylated sequence GUG (pGUG) whose binding of over 40% was comparable to or better than that of the tetranucleotide GUGU or UGUG (Figure 3C). Consistent with a minimum of three phosphates, no binding was observed for the phosphorylated mono- and dinucleotides. The fact that the trinucleotide is the minimum length for RNA binding to an amyloid is interesting for its correspondence to the length of a codon. To get an idea of the affinity of these interactions, we measured the binding for the oligonucleotides pGUG, pGUGU, pGUGUG, [GU]_3_, and [GU]_3_G with 50 μM peptide **7** using a range of RNA concentrations from 5–200 μM (Figure S7). The data were fit to a single binding site, and the results presented in Figure S7 reveal a strong correlation between the affinity and the length of the oligonucleotide. The *K*_d_ values for the series pGUG, pGUGU, and pGUGUG are 82 μM, 1.3 μM, and 75 nM, respectively, while that of hexanucleotide [GU]_3_ (0.85 μM) lies between that of the phosphorylated tetra- and pentanucleotides, decreasing to 49 nM for the heptanucleotide [GU]_3_G. For each additional nucleotide, the binding increases by more than an order of magnitude, which confirms our assumption of an avidity-enhanced RNA–amyloid interaction. Interestingly, phosphorylated oligonucleotides bind better than their longer, nonphosphorylated counterparts bearing the same number of phosphates (pGUG vs [GU]_2_ and pGUGUG vs [GU]_3_) (Figures 3 and S7). This could be due to the extra charge density on the 5′ phosphates contributing more to the binding than the additional nucleotides. In the context of the relatively low specificity of the codon–anticodon interaction,^27,28^ it is noted that the μM affinity of this potentially prebiotic interaction between the peptide amyloid and the RNA trinucleotide is at least 1–2 orders of magnitude stronger than the expected affinity between a complementary pair of trinucleotides.^29,30^ Based on the fraction bound at saturation, the binding stoichiometry for the heptanucleotide is three peptides per RNA molecule, while for the trinucleotide it is one to one.

Having observed significant sequence selectivity for the RNA heptanucleotide sequences (Figure 2C), we also investigated how the sequence composition of the trinucleotide affects its binding. In addition to pGUG, the other 5′-phosphorylated pGNG trinucleotides (pGCG, pGAG, and pGGG) as well as the other poly-N sequences (pAAA, pCCC, and pUUU) were measured in the binding assay with peptides **7** and **9**. The results shown in Figure S8 indicate a strong dependence on the RNA sequence for binding, even in the context of a codon-sized trinucleotide. Here, the lack of binding of pAAA and pCCC to peptides **7** and **9** could be explained by the expected positive charge character on adenine (p*K*_a_ ≈ 3.7) and cytosine (p*K*_a_ ≈ 4.3) at pH 3. In line with this argument, we find that at pH 5, these same two trinucleotides exhibit sequence-selective binding to the V and A variants of peptide set **S7** from Table S1 (Figure S8C).

Considering the significantly better binding of pGGG to all of the tested peptide amyloids, and despite sodium being the only metal cation added to the system, we questioned whether the poly-G sequence may be supporting the formation of a quadruplex. However, we could not detect any temperature dependence on the UV absorption spectrum of pGGG that would indicate that an oligomerization occurs. Also, we measured the diffusion coefficients of pGGG and pGAG by diffusion-ordered NMR spectroscopy (DOSY) and found them to be too similar to be consistent with a difference in oligomerization state; assuming roughly spherical molecules, tetramerization would lead to an increase in the radius by a factor of ∼1.59 (4^(1/3)^) with an inversely proportional decrease of the diffusion coefficient by a factor of 0.63. However, the observed diffusion coefficients ((5.37 ± 0.23) × 10^–10^ m^2^/s for pGGG and (5.19 ± 0.52) × 10^–10^ m^2^/s for pGAG) have a ratio of 1.03 (Figure S9).

**Figure 4:**
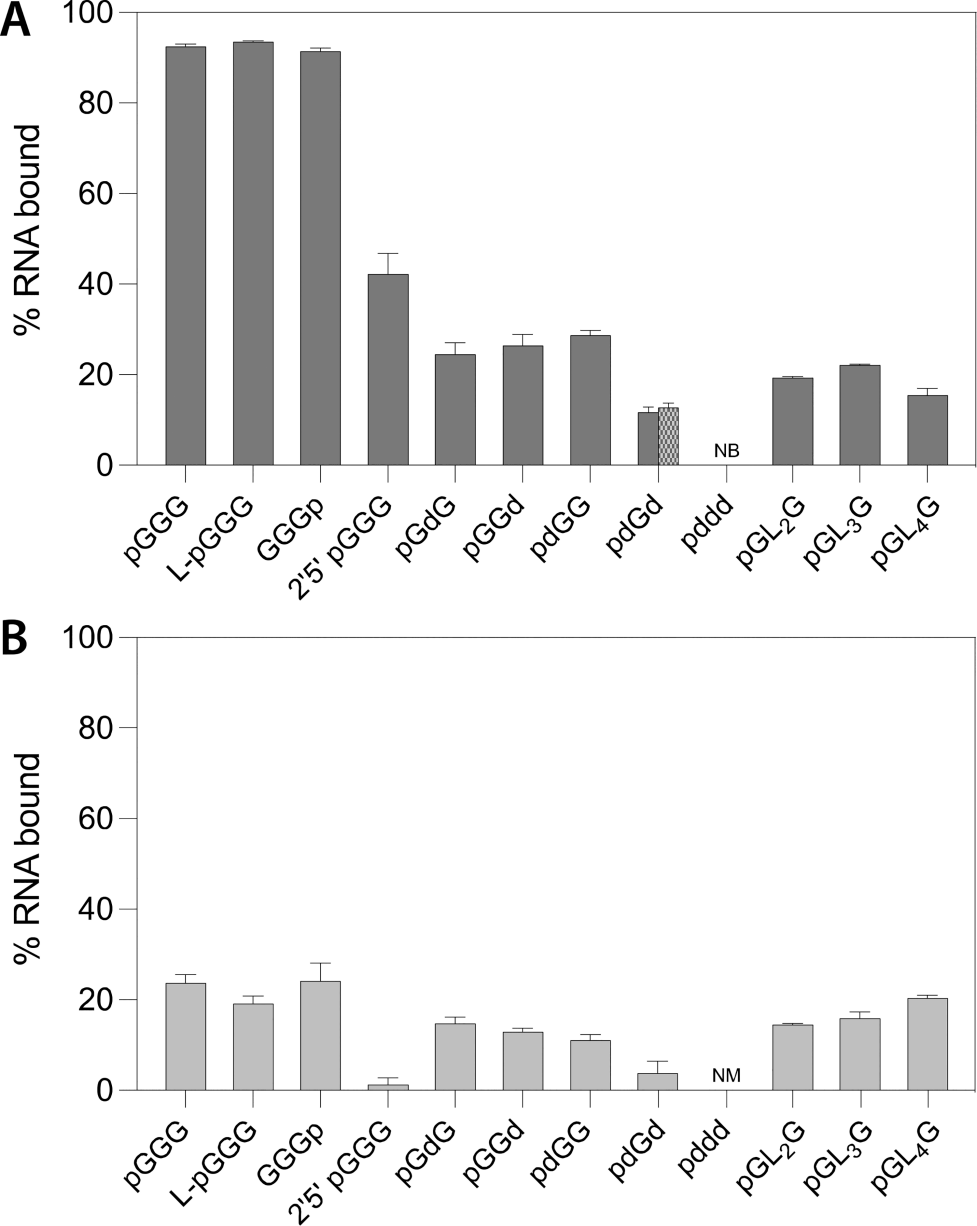
Structural determinants of RNA–amyloid interactions. (A) Binding of the 5′-phosphorylated RNA trinucleotides (or their analogues) to peptide **7**. l-pGGG is the mirror-image isomer of pGGG, while GGGp is phosphorylated at the 3′ position instead of the 5′ position. The 2′,5′-pGGG has 2′–5′ phosphodiester linkages. The “d” in the sequences denotes a deoxyribose without a nucleobase. pdGd binding was measured by both HPLC and ^1^H NMR (checkered pattern). NB = no binding was observed for pddd to the peptide based on ^1^H NMR. The “L_*n*_” in the sequences denotes linkers of *n* = 2–4 carbons (i.e., the linear 1,*n* diols of ethane, propane, or butane) in place of a nucleotide. (B) Same RNA analogues as in A binding to peptide **9** (NM = not measured for pddd). The assays were performed at room temperature and pH 3 with 100 μM peptide and 25 μM RNA. Errors are given as the standard deviation of two completely independent assays.

**Figure 5:**
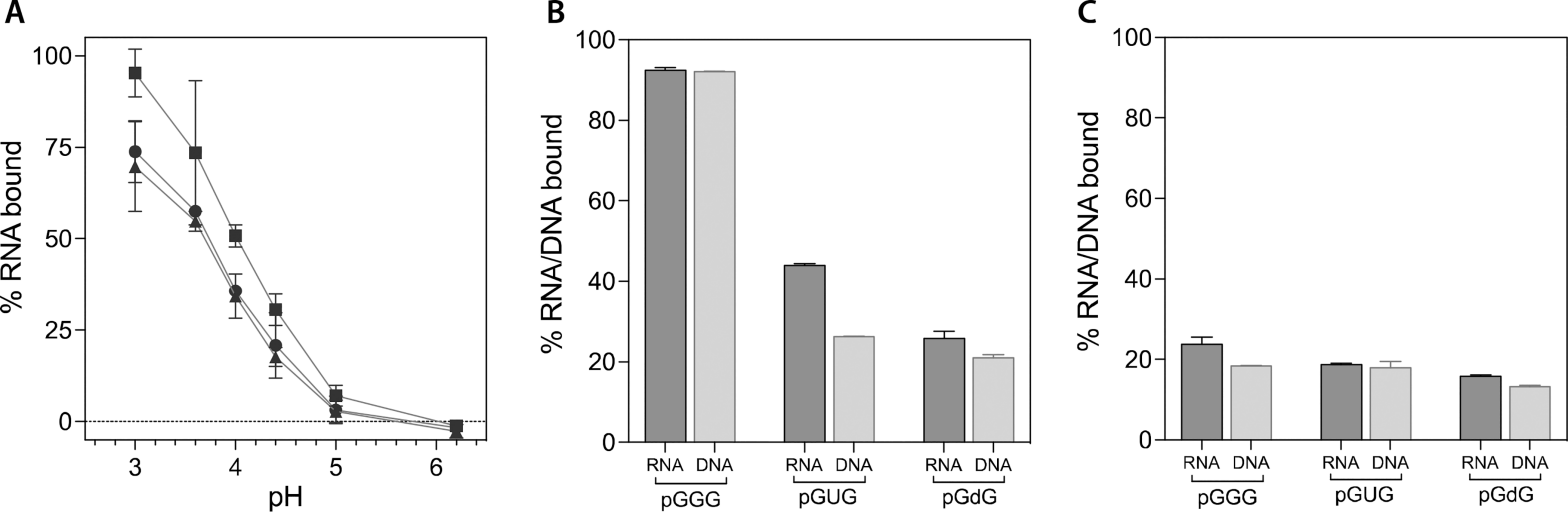
Differential amyloid binding of DNA and RNA. (A) The pH-dependent interaction of RNA 7-mers [GU]_3_G (squares) and 2′-O-methylated [GU]_3_G (circles) and DNA 7-mer d[GT]_3_G (triangles) with peptide **7**. The assay was performed at room temperature in a citrate–phosphate buffer with 100 μM peptide and 50 μM oligonucleotide. (B) Binding of phosphorylated RNA and DNA trinucleotides (and their analogues) to peptide **7**. The assay was performed at room temperature in a citrate–phosphate buffer at pH 3 with 100 μM peptide and 25 μM oligonucleotide. (C) Same RNA analogues as in B binding to peptide **9**. The “d” in the sequences denotes a (deoxy)ribose without a nucleobase. Errors are given as the standard deviation of two completely independent assays.

To probe the role of the RNA structure in the binding interaction, we assayed a series of RNA and RNA analogues of the pGGG trinucleotide for binding to peptides **7** and **9**. First, we tested the three isomers of pGGG. The mirror-image stereoisomer (with l-ribose) displayed very similar binding, as did the 3′-phosporylated structural isomer. However, the 2′–5′ linked structural isomer bound significantly less to the amyloids, in particular to the peptide **9** amyloid, for which the binding was negligible (Figure 4). This may indicate that the spacing between the atoms in the RNA is more important than their relative symmetry. To probe the role of the nucleobases in the interaction, we tested a series of trinucleotides in which one or more nucleotides was replaced with an abasic deoxyribonucleotide (pGdG, pGGd, pdGG, pdGd, pddd). In all cases, the amount of binding correlated with the number of bases present, with the completely abasic pddd having no detectable binding (measured by ^1^H NMR). Next, we probed the spacing and flexibility between the terminal nucleotides with a series of diol linkers of 2–4 carbons in length (pGL_2_G, pGL_3_G, pGL_4_G). The three different pGL_*n*_G RNAs all had a similar percentage bound to the amyloids and were in a range similar to that of pGdG (single baseless RNA). The above data, summarized in Figure 4, indicate that in addition to the phosphate backbone, all three bases can have a significant impact on the RNA–amyloid interaction. Finally, we probed the role of the 2′ substituent via a comparison of the DNA and 2′-O-methylated RNA backbones in a heptanucleotide. The pH dependence of the binding of the methylated RNA and DNA heptanucleotides to the peptide **7** amyloid was similar, with both displaying a weaker binding than the [GU]_3_G RNA (Figure 5A). While the DNA heptanucleotide d[GT]_3_G differs by the presence of the thymine methyl groups, these did not appear to have a large effect on the binding. We also explored the differences between the binding of the DNA and RNA trinucleotides (pGGG, pGUG**)** and their analogue (pGdG) to peptides **7** and **9** and found similar observations of better RNA than DNA binding (Figure 5). This is also supported by affinity measurements with peptide **7** (Figure S7), for which the DNA trinucleotide pGUG had a *K*_d_ of 117 μM and the RNA trinucleotide pGUG had a *K*_d_ of 82 μM.

**Figure 6:**
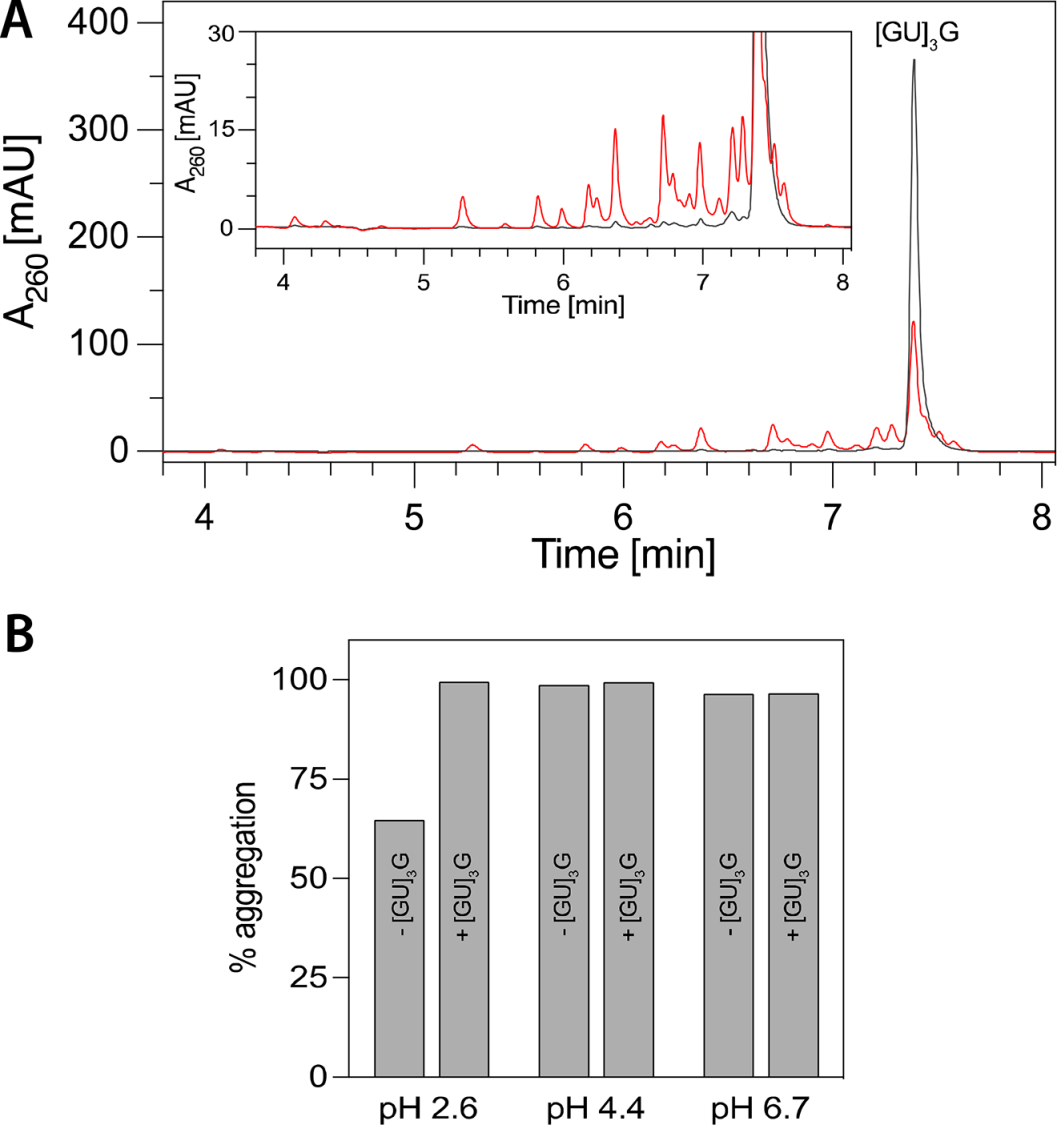
Mutually stabilizing effect of RNA–amyloid interactions. (A) RNA protection by amyloids. HPLC chromatograms are given for samples of [GU]_3_G (red) and [GU]_3_G in the presence of peptide **7** (black). The samples were incubated at room temperature in a citrate–phosphate buffer at pH 2.6 for 36 days. The peptide concentration was 100 μM, and the RNA concentration was 20 μM. Similar protection was observed at 50 °C for 16 h (Figure S10). (B) Amyloid stabilization by RNA. The aggregation of peptide **7** alone and in the presence of [GU]_3_G at different pH values is represented as bars in the plot. The assay was performed at room temperature in a citrate–phosphate buffer with 100 μM peptide and 50 μM RNA by a quantitative HPLC analysis of the soluble fraction.

In the context of prebiotic interactions between RNA and amyloids, symbiotic relationships could be important in selection/survival processes. Therefore, we compared the stability of RNA in the presence and absence of amyloids by incubating them for 36 days at room temperature or 16 h at 50 °C. The results presented in Figure 6A and Figure S10 reveal a striking enhancement of the [GU]_3_G RNA stabilization against hydrolytic degradation when in the presence of the peptide **7** amyloid, suggesting that a prebiotic environment like heated rock pores, in which convection and thermophoresis lead to thermal cycling, could select for RNAs that are bound to and protected by amyloids.^31^ On the other hand, we also found that the presence of [GU]_3_G enhanced the aggregation of peptide **7** at pH 2.6, which at this pH did not completely aggregate on its own, likely due to the protonation of the glutamate residues disrupting the electrostatic interactions with the lysine residues (Figure 6B).

**Figure 7:**
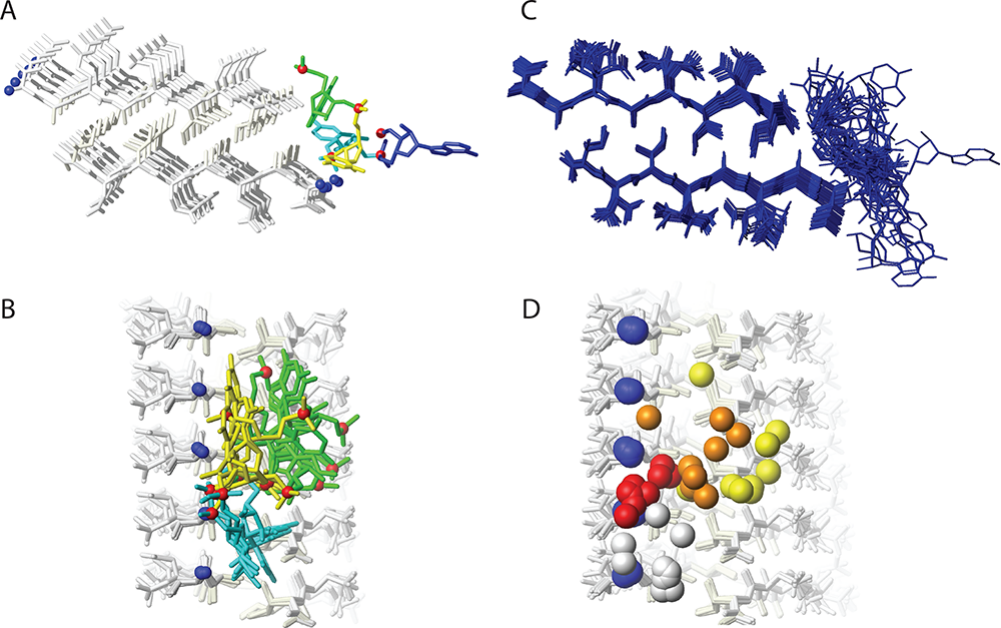
3D solid-state NMR structure of the RNA–peptide amyloid complex. The 3D solid-state NMR structure of the peptide amyloid VAQAQINI-NH_2_ with the RNA pGUCAp is shown (PDB 8PXS). (A) View down the long fibril axis of the peptide amyloid, displayed as 2 × 5 molecules of the peptide with the hydrophobic side chains in light yellow, the hydrophilic side chains in gray, the backbone in white, and the N-termini as blue spheres, illustrating the class 1 steric zipper with a hydrophobic core. The RNA is color-coded with G in green, U in yellow, C in cyan, and A in blue, and the phosphates are shown as red spheres. (B) Structural bundle of the 10 conformers with the lowest target function from the structure calculation. (C) Side view of the RNA–amyloid complex showing four conformers representing the RNA binding site (the A4 nucleotide and 3′ phosphate moieties are not displayed for clarity). (D) Side view illustrating the electrostatic interaction between RNA phosphates, colored from 5′ to 3′ in yellow, orange, red, and white (3′ terminal phosphate not displayed), and the N-terminus of the peptide in blue.

In order to gain a deeper insight into the determinants of amyloid–RNA interactions, we undertook the structural characterization of one interacting pair by solid-state NMR. In the study of the aggregated states of peptides and RNA, solid-state NMR has many advantages over other methods, but it requires that the peptide and the RNA sequences have low spectral overlap in order to be able to resolve the resonances. For this task we chose the peptide VAQAQINI-NH_2_ and the ribonucleotide pGUCAp bearing both 3′ and 5′ phosphorylation in order to increase the affinity. Using a combination of unlabeled, specifically labeled, and uniformly ^13^C-,^15^N-labeled peptides and site-specific ^13^C-labeled RNA, we collected a series of 2D ^13^C,^13^C and ^13^C,^31^P solid-state NMR spectra (Figures S11–S15 and Tables S3–S5) for sequential assignment and structure calculation (following procedures described in detail in the Supporting Information). The spectra with intermolecular cross peaks between the RNA and the protein indicate an interaction of the RNA with both the N- and C-termini of the peptide. Both the phosphates as well as the bases of G1, U2, and C3 interact with the amyloid via residues Val1 at the positively charged N-terminus or Ile8 on the C-terminus of the peptide. Sufficient distance and angular restraints could be collected from the spectra (Table S2) for an RNA–peptide amyloid complex structure determination, yielding a well-defined peptide amyloid structure with an overall root-mean-square deviation (RMSD) of 0.8 Å and bound to a less well-defined RNA molecule having an overall RMSD of 1.5 Å (Table S2). The 3D structure of the peptide amyloid is composed of two parallel β-sheets that interact with each other face-to-face (i.e., C_2_ symmetry), burying a hydrophobic core comprised of residues Ala2, Ala4, Ile6, and Ile8 and forming a class 1 steric zipper^32^ (Figure 7). This yields an amyloid fibril with two identical edges composed of the positively charged N-terminus, the Val1 side chain, the solvent-exposed face of the steric zipper motif composed of the side chains of Ala2 and Ile8, and the peptide C-terminal amide. It is at these edges that the RNA is bound in the complex structure. In particular, the 5′ phosphate of nucleotide C3 interacts with the positively charged N-terminus. In addition, the other three phosphates, with the exception of the phosphate at the 3′ end, are in proximity to the N-terminus, illustrating the electrostatic nature of the interaction as suggested by the interaction studies in Figures 2B, 3, and 4. In particular, the structure explains the lack of interaction in the absence of a positively charged N-terminus and the importance of at least three phosphates for significant binding. In addition, the bases of the first three nucleotides G1, U2, and C3 interact with the exposed hydrophobic edge composed of Ala2 and Ile8 and have the potential to make hydrogen bonds with the amidated C-terminus. The relevance of the nucleobases for RNA–amyloid interactions has been demonstrated by the experiments detailed in Figure 4, for which no measurable binding was observed in a baseless trinucleotide analogue, and for which the deletion of one or two nucleobases reduced the binding several-fold. Finally, the structure explains how the interaction is relatively insensitive to the composition of the backbone, be it ribose, deoxyribose, or linker (Figures 4 and 5), as the sugars are not part of the interface. The measured affinity for pGUCAp binding to VAQAQINI-NH_2_ of 2 μM (Figure S7G) as well as the NMR data indicate that the interaction of the RNA with the peptide amyloid spanning about 2–3 peptides on the edge of the fibril is rather dynamic in nature. The broad phosphate NMR cross peaks in Figure S11 indicate the presence of structural plasticity and a weak interaction. Considering the structural data for the pGUCAp/VAQAQINI-NH_2_ complex, with binding modes of the electrostatic interaction between the RNA phosphates and the peptide N-terminus and the mostly hydrophobic interactions between the bases and the edge of the hydrophobic core of the peptide amyloid, a wide range of interactions could be expected, with the calculated structure providing some snapshots.

With the aim to establish codon (anticodon)–amino acid specificity, we pursued a more detailed investigation of the sequence-specific nature of the RNA trinucleotide–amyloid interactions. For this, we analyzed the interactions between phosphorylated trinucleotides with the sequence pGNG or pGNC (N = G, A, C, or U) and 18 sets of amyloidogenic peptides for which one or more positions were systematically varied as Ala, Val, Gly, and Asp/Glu (Table S1). Considering the RNA–amyloid structure presented above, we mostly chose the variable amino acid positions to be located either at the N-terminus with up to three repeats or at the C-terminus, with some at expected solvent-exposed positions.

**Figure 8:**
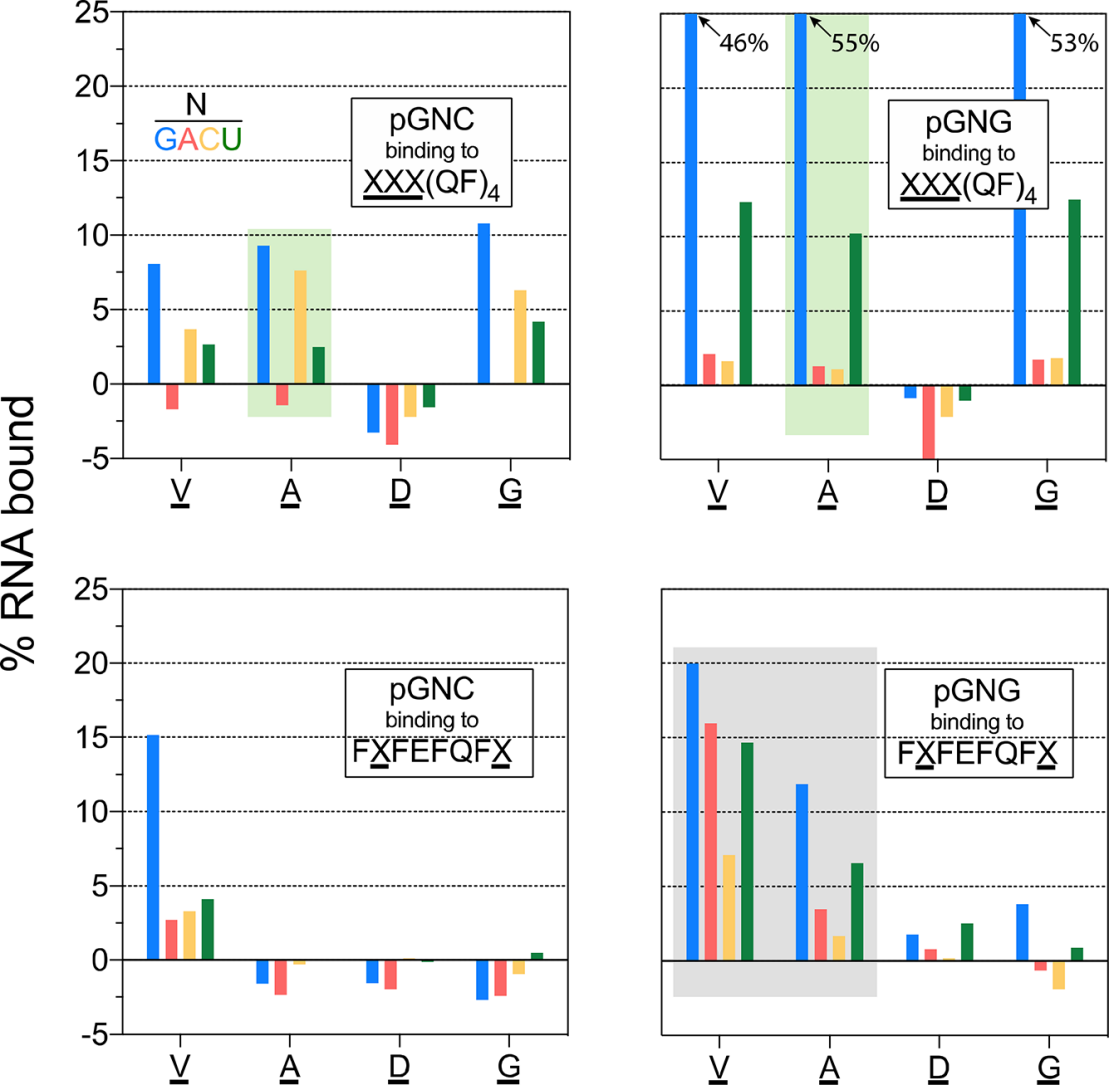
Sequence-selective RNA trinucleotide–amyloid interactions. The percent bound of each RNA of an RNA trinucleotide pool (either pGNG or pGNC with N = G in blue, A in red, C in yellow, or U in green) to one of four amyloids (either XXX(QF)_4_–NH_2_ or FXFEFQFX with X = V, D, A, or G, see Table S1). Each panel represents four experiments, in each of which one pool of four trinucleotides was bound to one of four peptide amyloids. The green shaded regions highlight the sensitivity of a particular amyloid (AAAFQFQFQFQ) to the second and third nucleotides of the RNA trinucleotide. The gray shaded region highlights the sensitivity of the RNA to the sequence of the amyloid. See Figure S16 for more results from a detailed screen on RNA–amyloid selectivity under various experimental conditions. Weak binding can also appear as a negative percent bound, which can be attributed to small errors in peak integration as well as peptide amyloid competition with the RNA for nonspecific binding to the walls of the assay tube.

The results presented in Figures 8 and S16 indicate that binding to a particular amyloid is generally higher for pGNG trinucleotides compared to pGNC; however, in both RNA sets, the identity of the central nucleotide can have a significant impact on the interaction. In the binding assay of pGNG with the AAA(QF)_4_ peptide (shaded green in Figure 8), the strength of the interaction follows the trend of G > U > A ≈ C for the central nucleotide, while for pGNC, this trend becomes G > C > U > A. Conversely, on the peptide side, selectivity at the second position of pGNG is entirely different for the Val and Ala variants of FXFEFQFX (shaded gray in Figure 8). The binding data for the entire set of 74 amyloids and eight trinucleotides are presented in Figure S16, with the selectivity highlighted as heat maps of the log of the ratio of the binding. Despite the large range of peptide sequences tested, we were not able to identify any general sequence determinants on an amyloid that would make it selective for a particular RNA sequence. Still, the data demonstrate that amyloids and codon-sized RNA can have selective interactions, which is in line with the large number of charge-based and hydrophobic contacts in the structure of the complex that we determined (Figure 7). Finally, the data in Figure S16 show that the sequence-selective RNA–amyloid interaction can be modulated depending on the assay conditions. Changes in temperature, pH, and salt concentrations may alter the physicochemical properties of the amyloid and the nucleobases, eventually affecting the electrostatic interactions.

In summary, we have found that small RNAs can bind to peptide amyloids in a sequence-dependent manner and that such interactions could be mutually beneficial for the RNA and the peptide by stabilizing the amyloid structure and reducing the extent of RNA hydrolysis. We have identified the important elements of the interaction, namely, a minimum of three ribonucleotides in which three phosphates and three nucleobases contribute to binding. While there are many theories on the origin of the genetic code, they all must be able to explain the self-evident fact that at one point, specific interactions developed between RNA and amino acids. Thus, our finding that small, codon-sized RNA molecules bind peptide amyloids through both nonspecific electrostatic interactions between the phosphate backbone of the RNA and cationic groups on the peptide amyloid (such as the N-terminus) as well as sequence-specific interactions via the nucleobases of the trinucleotide suggests a mechanism by which RNA–amyloid interactions could support the origin of a genetic code. Such a mechanism is interesting due to the simplicity of both the RNA and peptide species involved and suggests that the initial specificity in the genetic code could have been established before RNA molecules that are large enough to bind to amino acids existed. Considering the plausible prebiotic nature of peptide amyloids,^9,33^ it is tempting to assume that peptide amyloids and RNA developed symbiotic interactions before more life-like systems developed. There are still more questions than answers, but our results provide an alternative pathway for the origin of the genetic code that is in line with the stereochemical hypothesis which posits that the genetic code reflects the affinity between the amino acid residues and their codons (or anticodons), with the physical association between them playing a role in the primordial soup before the translation machinery developed.^34,35^ There are several lines of evidence that support this hypothesis, including early work that demonstrated codon–amino acid interactions,^36^ the physical proximity between codons and their cognate amino acid residues in the ribosome and other RNA binding proteins,^37,38^ and the extremely small probability that pure chance has led cognate codons to appear at such a high frequency in the amino acid binding regions of natural amino acid binding RNAs and artificial aptamers.^39,40^ It has been argued that the codon specificity for the four most prominent prebiotic amino acids (i.e., Gly, Asp, Val, Ala) could be encoded by the central nucleotide of a GNC code.^41^ In the context of our data, this suggests that nature’s selection of a codon length of three nucleotides was in order to generate sufficient avidity in the defining interactions of RNA with amino acids (e.g., between peptide amyloid and RNA) because a dinucleotide codon would have been sufficient to code for the few amino acids in the early phase of life. Finally, it is worth noting that amyloids, with their periodic structure and well-defined surface, now have the proven potential to increase the local concentration and order of nucleotides in an otherwise dilute disordered system. The sequence-selective nature of said interactions as well as the catalytic ability of the amyloid could have promoted the synthesis of distinct and longer ribonucleotides, possibly an important step for the evolution of catalytic RNAs.

## Supporting Information Available

Materials and methods; additional experimental details; characterization of the amyloids by FTIR, TEM, and HPLC; RNA–amyloid affinity measurements; additional RNA–amyloid binding data; DOSY spectra of trinucleotide RNA; additional RNA protection data; ssNMR spectra; RNA–amyloid specificity plots; and data collection parameters and structure statistics for ssNMR-based structure determination (PDF)
