# Supplementary material for: An Analysis of Nucleotide–Amyloid Interactions Reveals Selective Binding to Codon-Sized RNA
Source: J Am Chem Soc. 2023 Oct 2;145(40):21915–24. doi: 10.1021/jacs.3c06287 (PMC10571083; doi:10.1021/jacs.3c06287)
Supplement: Supplementary file 1 — ja3c06287_si_001.pdf [file ja3c06287_si_001.pdf]

Supporting Information for

An analysis of nucleotide-amyloid interactions reveals selective binding to codon-sized RNA

Saroj K. Rout[1], Riccardo Cadalbert[1], Nina Schröder[2], Julia Wang[2], Johannes Zehnder[1], Olivia Gampp[1], Thomas Wiegand[2,3], Peter Güntert[1,4,5], David Klingler[6], Christoph Kreutz[6], Anna Knörlein[7], Jonathan Hall[7], Jason Greenwald[1] and Roland Riek[1*]

[1]Institute of Molecular Physical Science, ETH Zürich, 8093 Zürich, Switzerland
[2] Institute of Technical and Macromolecular Chemistry, RWTH Aachen University, 52074 Aachen, Germany
[3] Max Planck Institute for Chemical Energy Conversion, 45470 Mülheim/Ruhr, Germany
[4]Institute of Biophysical Chemistry, Goethe University, 60438 Frankfurt am Main, Germany
[5]Department of Chemistry, Tokyo Metropolitan University, Hachioji 192-0397, Japan
[6]Institute of Organic Chemistry and Center for Molecular Biosciences Innsbruck (CMBI), Universität Innsbruck, 6020 Innsbruck, Austria
[7]Institute of Pharmaceutical Sciences, ETH Zürich, 8093 Zürich, Switzerland

Keywords: amyloids, RNA, genetic code, origin of life

*correspondence to: roland.riek@phys.chem.ethz.ch

Peptide manipulations

The unlabelled peptides, purchased from GLS China, were synthesized using standard Fmoc chemistry and used as either pure or crude compounds. The crude peptides were purified by reverse phase HPLC on a Kinetex C18 5µm 10x250mm column (Phenomenex) in CH_3CN/H_2O/TFA (peptides 4 and 7) or CH_3CN/H_2O/TEAA (peptides 5, 6, 8, 9, 10 and 11) solvent systems. The quantitation of the peptides was performed by reverse phase HPLC and was based on their calculated extinction coefficient at 214 nm[1]. Pure peptides were stored in lyophilized aliquots. The different specifically $^{13}C/^{15}N$ labeled variants of the VAQAQINI-NH_2 peptide including uniform $^{13}C/^{15}N$-labeling, as well as V^{15}N-AQAQI^{13}C-I-NH_2 and V^{13}C-AQAQI^{15}N-I-NH_2 were synthesized on an Applied Biosystems 433 A automated batch peptide synthesizer. The product purities were controlled by reversed-phase analytical HPLC.

RNA oligonucleotides/analogs

The RNA oligos and analogs, with the exception of **pdGd, pddd** and isotopically-labelled **pGUCAp** were purchased from ChemGenes Corporation (USA). The d**[GT]$_3$G** DNA was purchased from Microsynth AG (Switzerland). For **pdGd** and **pddd**, the synthesis was performed in-house on an MM12 synthesizer (BioAutomation Corp., Irving, TX) on a 50 nmol scale with 2′-O-TBDMS-guanosine phosphoramidite (Thermo Fisher), the dSpacer Phosphoramidite (GlenResearch, Sterling, VA), and the DNB (dinitrobenzhydryl) phosphoramidite using a 500 Å UnyLinker support (ChemGenes, Wilmington, MA). The DNB phosphoramidite and the oligoribonucleotides were synthesized as previously described by Pradere *et al.* [2].

Oligonucleotides were deprotected from support using 1 bar gaseous methylamine at 65 °C for 1.5 h. For the **pdGd**, TBDMS protection group was removed by adding a freshly prepared mixture of 1-N-methyl-2-pyrrolidone:TEA:HF·3 TEA 6:3:4 (130 µL) and incubation at 70 °C for 90 min. The DNB protection group was removed by UV irradiation at 365 nm for 15 min on a transilluminator. In all cases, except **pddd**, the oligonucleotides, were obtained in desalted form and then purified by reverse phase HPLC on a Kinetex C18 5 µm 10x250 mm column (Phenomenex) in a CH_3CN/H_2O/TEAA solvent system and quantitated by their extinction coefficient at 260 nm. Pure oligos were then lyophilized and dissolved in water at a concentration of more than 1 mM and stored at -80 °C and then diluted to different buffers as required. For **pddd** the dinitrobenzhydryl (DNB) protecting group was left in place to facilitate HPLC purification and quantitation. It was removed by UV irradiation at 365 nm for 5 min on a transilluminator and then passing the sample over a C18 SPE column. **pddd** concentration

was determined by the ^{1}H-NMR signals of the ribose compared to the corresponding signals in the uncleaved DNB-**pddd** sample.

Isotopically-labelled RNA synthesis

The isotopically-labelled **pGUCAp** RNA [5'-p-(^{13}C8-G)(^{13}C6-U)(^{13}C6-C)(^{13}C8,^{13}C2-A)-3'-p] was produced using in-house synthesized [5-D-6-^{13}C] uridine, [1,3,4-$^{15}N_3$-5-D-6-^{13}C] N^{4}-acetyl-cytidine, [8-^{13}C] N^{2}-iBu-guanosine, and [2,8-$^{13}C_2$] N^{6}-acetyl-adenosine building blocks with 2'-O-TBDMS protecting groups. The 5'-terminal phosphate was introduced using an in-house synthesized O-DMT-2,2'-sulfonyldiethanol phosphoramidite. 100 mg of phosphoramidite was dissolved in 1 ml of absolute acetonitrile. Controlled pore glass 3'-phosphate RNA solid support (1000 Å pore size, average loading 98 µmol/g, *GlenResearch,* USA) was used to produce the RNA on a *K&A Labs* H6 synthesizer using a self-written RNA synthesis cycle. Amidite and BTT activator solutions (5-benzylthio-*1H*-tertrazole, 250 mM in absolute acetonitrile) were dried over freshly activated molecular sieves (3 Å) for at least 36 hours before use. The following reagent solutions were used: *Cap A:* acetic anhydride/2,6-lutidine/tetrahydrofuran 1/1/8 (v/v/v). *Cap B:* N-methylimidazole/tetrahydrofuran 14/86 (v/v). *Oxidation solution:* 500 mg of iodine dissolved in 70 ml tetrahydrofuran, 20 ml pyridine and 10 ml water. *Detritylation solution:* 4% dichloroacetic acid in 1,2 dichloroethane. Following the RNA synthesis, the resin was dried for 1h under high vacuum. For alkaline deprotection 1 ml each of ammonium hydroxide (28-30%) and aqueous methylamine (40%) were added to the solid support. The reaction tube was incubated at 37 °C for 5 hours. During this time, it was regularly agitated. Then, the solid support was filtered off and washed three times using a mixture of tetrahydrofuran and water (1/1). The combined liquid phases were evaporated to dryness and the remaining white residue dried *in vacuo* for 1 hour. For 2'-O-TBDMS deprotection the residue was dissolved in 300 µl anhydrous dimethylsulfoxide. 375 µl triethylamine trishydrofluoride was added and the mixture incubated at 37 °C for 16 hours. Upon completion, the reaction was quenched using 3 ml of quenching buffer (*GlenResearch*, USA). The mixture was then applied to a HiPrep 26/10 desalting column (*GE Healthcare*, Austria) using an ÄKTA start system (*GE Healthcare*, Austria). The RNA was eluted using HPLC-grade water. The fractions containing the desired RNA (UV detection at 254 nm) were collected in a 50 ml round bottom flask and evaporated to dryness. The residue was dissolved in 1 ml HPLC-grade water and stored at -20 °C until needed. The quality of the RNA was checked via anion-exchange chromatography over a Dionex DNAPac PA-200 column (4x250

mm; *Eluent A:* 25 mM Tris.HCl, pH 8.0, 20 % (v/v) acetonitrile, 10 mM sodium perchlorate; *Eluent B:* 25 mM Tris.HCl, pH 8.0, 20 % (v/v) acetonitrile, 600 mM sodium perchlorate).

Fibrillization

Lyophilized aliquots of pure peptides were dissolved in water (peptides 4, 5, 7, 8, 9, 10 and 11 in Table 1, and peptide series 1-5 in Table S1 at pH ~ 8.5-9) or DMSO (peptides 1, 2, 3 and 6 in Table 1 and peptide series 6-18 in Table S1) at a concentration of more than 5 mM. The stock was then diluted to typical concentration of 200 µM (unless otherwise stated) in the citrate-phosphate buffer of the desired pH and incubated overnight at room temperature in a thermomixer (Eppendorf) with agitation at 1000 r.p.m. The fibrillization was monitored by Fourier transform infrared spectroscopy and HPLC-based supernatant analysis.

Fourier transform infrared spectroscopy

The peptide aggregates in citrate-phosphate buffer were centrifuged at 25,000 g, and the insoluble material (pellet) was washed in 10 mM HCl and centrifuged again. The pellet was resuspended in 5 µl 10 mM HCl and applied to a diamond ATR cell on a Bruker Alpha FTIR spectrometer. The samples were air-dried before measuring their spectra with 32 scans and a resolution of 2 cm^{-1}. The buffer exchange was important as the strong citrate-phosphate buffer absorbance overlaps with the relevant portion of the peptide spectrum.

Transmission electron microscopy

The samples prepared as for the IR measurements were applied directly to the negatively glow-discharged carbon-coated copper grids, washed with water and stained with phosphotungstic acid. The imaging was done on a FEI Morgagni 268 electron microscope.

Amyloid-RNA binding assays

Amyloid aggregates and RNA/DNA oligos were mixed in an Eppendorf DNA LoBind® tube at the desired concentrations in the citrate-phosphate buffer at different pH. After overnight incubation of the mixture, the tubes were centrifuged at 25,000g for 1h. The supernatant was collected, and reverse-phase analyses in a CH_3CN/H_2O/TEAA solvent system were performed on a BioZen C18 2.6µm 4.6 x 150mm column (Phenomenex) connected to an Agilent 1200 HPLC system equipped with an autosampler and diode array detector. The samples injected onto the column are resolved using a linear gradient of acetonitrile (1-15%) at a flow rate of 1

ml/min. The analyses related to **pGUCAp** were done via anion exchange on a LUNA NH_2 5µm 4.6 x 150mm column (Phenomenex) using H_2O/TEAA/$CH_3CO_2NH_4$ buffers. The concentration of each oligonucleotide that remained soluble in the amyloid-RNA mixture was calculated using the HPLC peak areas and extinction coefficients at 260 nm. These values were then subtracted from the total oligos in the assay to determine the amount of the RNA bound to the amyloid.

DOSY NMR

1D ^{1}H NMR measurements of 50 µM **pGGG** and **pGAG** in citrate-phosphate buffer pH 3 were carried out with a diffusion-filtered pulse sequence comprising two pairs of pulsed field gradients bracketing a diffusion time of 120 ms on a Bruker Avance III HD 600-MHz spectrometer (Fällanden, Switzerland) equipped with triple resonance TCI CryoProbe (Bruker). The gradient strength was varied from 5-95% (2.4-45.8 Gauss/cm) in steps of 5%. The data were analyzed in the Dynamics Center software (Bruker) by fitting the peak areas for several groups of resonances to the function: $f(G) = I_0 e^{\left(-DG^2\gamma^2\delta^2\left(\Delta - \delta/3\right)\cdot 10^4\right)}$ with γ = 26752 rad/s·Gauss, δ = 2 ms and Δ = 120 ms.

Solid-state NMR structure determination of the complex between the VAQAQINI-NH_2 peptide amyloid and the RNA pGUCAp

Amyloids of VAQAQINI-NH_2 were prepared in citrate-phosphate buffer pH 3 with uniformly labeled peptide (sample I), mixed unlabeled and uniformly labeled peptides in a ratio of 2.8:1 (sample II, "diluted" sample), mixed-labeled peptides V^{15}N-AQAQI^{13}C-I-NH_2 and V^{13}C-AQAQI^{15}N-I-NH_2 in a ratio 1:1 (sample III), uniformly labeled peptide amyloids in presence of unlabeled RNA pGUCAp (sample IV) and uniformly labeled peptide amyloids in presence of specifically ^{13}C-labeled RNA pGUCAp (sample V, as described above) in an approximate ratio of 10:1. Sample I-IV were filled by established procedures using home-built tools[3] in 3.2 mm rotors, while due to limited amounts of sample V, it was filled in a 1.9 mm rotor, as was a second sample of sample I.

The solid-state NMR experiments collected for these samples for both the sequential assignments and structure determination are summarized in Tables S3-S5 and in part shown in Figures S12-S15. For the peptide, solid-state NMR sequential backbone and side-chain resonance assignments were obtained by state-of-the-art 2D NMR experiments, first for the peptide amyloid sample alone and then for the complex with RNA. The assignment of the

specifically labeled ^{13}C of the RNA was established both by chemical-shift statistics (from the BMRB database) and the presence of inter-residue cross peaks between C8 of guanine and C6 of uracil, C6 of uracil and C6 of cytidine and an intra-residue cross peak C2-C8 of adenosine observed in the 450 ms DARR spectrum recorded on sample V. Three distinct ^{31}P chemical shifts were identified in the [^{13}C,^{31}P] CHHP 2D spectrum.[4-5] However, they are of ambiguous nature, since due to a limited signal-to-noise ratio for sample V no ^{13}C-^{31}P cross peak within the RNA was detected in a corresponding experiment (not listed in the Tables S3-S5). All of the amino acid residues were assigned. The chemical-shift values have been deposited in the BMRB data bank, accession number 34838.

The collection of restraints for the structure calculation started from the sequential assignment. Based on the sequential assignment the identification of the β-sheet secondary structure of residues 2-7 of the peptide amyloid could be identified and yielded angular restraints using the TALOS+ databank[6] within CYANA. Next, a [^{13}C,^{15}N]-Proton Assisted Insensitive Nuclei Cross Polarization (PAIN) experiment[7] was measured on the mixed sample III. The identification of ^{13}C-^{15}N peaks for Ala2 and Ile8 indicates the presence of an in-register parallel β-sheet yielding valuable inter-molecular hydrogen-bond restraints for the structure calculation. Next, distance restraints were collected from a 20 ms DARR spectrum of sample I as well as for the RNA peptide complex sample IV showing very similar spectra as the *apo* form. All these distance restraints were treated in the following as ambiguous in respect to whether they are of intra or inter-molecular nature. A 2D PAR of the mixed sample III allowed the unambiguous identification of the inter-sheet distances CB Ala2 – CA Ile8, CB Ala2 – CB Ile8, and CB Ala2 – CG1 Ile8. RNA peptide distance restraints were collected from ^{13}C-^{31}P and ^{13}C-^{13}C cross peaks in the corresponding 2D spectra from sample IV and from 2D 150 ms and 450 ms DARR of sample V, respectively. ^{13}C-^{31}P distance restraints were treated as ambiguous with respect to ^{31}P excluding the 3'-end phosphate group, since no distance restraints from adenine were detected which also shows very weak signals for its ^{13}C moiety. An unambiguous ^{13}C amyloid peptide assignment of both ^{13}C-^{31}P and ^{13}C-^{13}C cross peaks was limited by both the 1:7 binding stoichiometry between RNA and peptide (Figure S7) as well as the low level of bound RNA (Figure S11) leading to a low signal-to-noise ratio of these inter-molecular cross peaks when compared with the intra-amyloid cross peaks. Additionally, RNA-binding induced ^{13}C chemical-shift perturbations of the peptide amyloid resonances, which are clearly evident on cross peaks between the RNA and the peptide amyloid resonances, are obscured by the intense cross peaks of the peptide amyloids. With this set of restraints summarized in Table S2 a 3D structure composed of 2 × 5 VAQAQINI-NH_2 and one RNA

molecule pGUCAp was initiated with CYANA[8] using intra-amyloid distance restraints of 7 Å for cross peaks obtained from the 450 ms DARR spectrum, of 5 Å for inter-peptide cross peaks observed in a PAR spectrum on the mixed sample III and of 6 Å for amyloid-RNA cross peaks measured either in the ^{13}C-^{31}P CHHP spectrum of sample IV or a [^{13}C,^{13}C] 450 ms DARR of sample V. This yielded a collection of 52 intra-amyloid distance restraints per peptide and 29 RNA–peptide distance restraints. Symmetry of the peptide structures was imposed by dihedral angle difference restraints (to maintain an identical conformation for all 10 peptide molecules in the structure calculation) and restraints on the differences between symmetry-related distances (to maintain symmetric relative positions)[9]. Two additional artificial distance restraints were added for the final calculation: The 4 Å distance between CB of Ala4 to CG1 of Ile8 present in an initial structure calculation in absence of RNA and also present in the complex with RNA for structure calculations with lowest CYANA target function values. The target function (TF) is defined as the sum of squared restraint violations and thus a descriptor of how well the structure fulfills the experimental restraints with a low value corresponding to only few and small violations. Without this additional restraint, calculations often ended with a high target function, while including it yielded a target function that was consistently lower by roughly 50% despite the presence of the additional restraint. This restraint is thus considered only a tool to help the structure calculation to converge towards a minimum that satisfies the experimental data. The second artificial restraint is a short-range distance between the positively charged N-terminus of the peptide and an ambiguous ^{31}P of the RNA. A 3 Å distance limit was selected in the final calculation, but similar structures are obtained also with a value of 6 Å (not shown). This restraint reflects the binding studies discussed above with many distinct peptide amyloids and RNA entities suggesting a charge-charge interaction between the two moieties.

The final CYANA structure calculation resulted in a well converged structure with an average target function of 6.6 Å^2 for the final bundle comprising the 10 best conformers (i.e. with lowest TF) indicating the presence of a self-consistent data set. The coordinates have been deposited at the PDB under ID 8PXS. The RMSD of the peptide amyloid is 0.1 Å for the backbone and 0.8 Å for all non-hydrogen atoms. The RNA is much less well defined compared to the peptide amyloid yielding an overall heavy atom RMSD of 1.5 Å (Table S2). We noted that during the early stages of refinement, the RNA molecule was sometimes oriented upside down bound to the peptide amyloid but that these alternative structures are absent in the final structure calculation result.

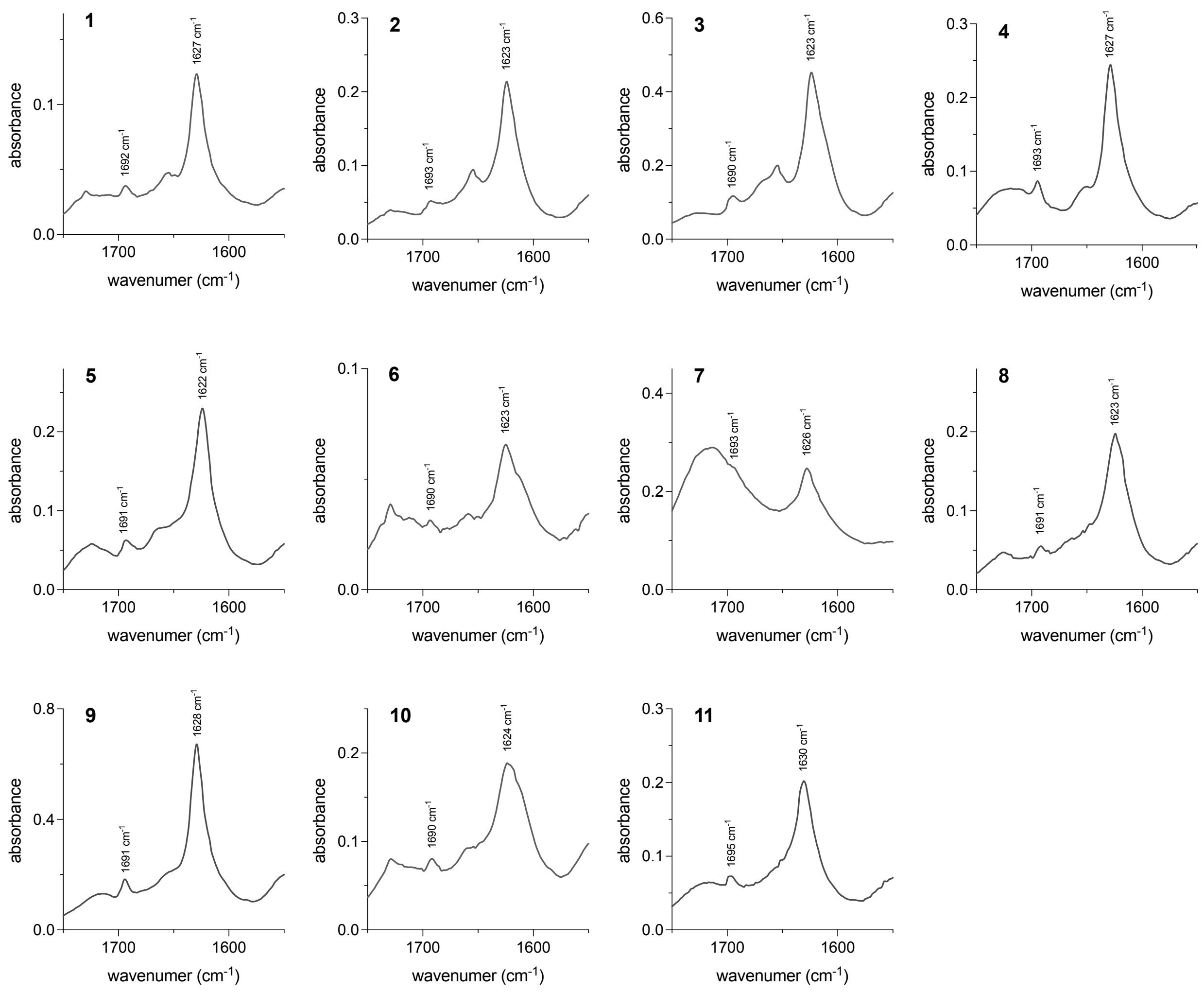

Figure S1. β-sheet aggregation of the peptides confirmed by FTIR spectroscopy. Each of the 11 peptides in this study (see Table 1) were characterized by FTIR. All of them exhibit the characteristic amide absorption near 1620-1630 cm^{-1} confirming the β-sheet aggregation and all have a smaller band near 1690 cm^{-1} indicating that the peptides form anti-parallel sheets in the fibers (as in the Figure 1 schematic). The peptide aggregates made at a concentration of 200 μM in citrate-phosphate buffer at pH 3 were centrifuged at 25,000 g, and the insoluble material (pellet) was washed in 10 mM HCl for the FTIR measurements.

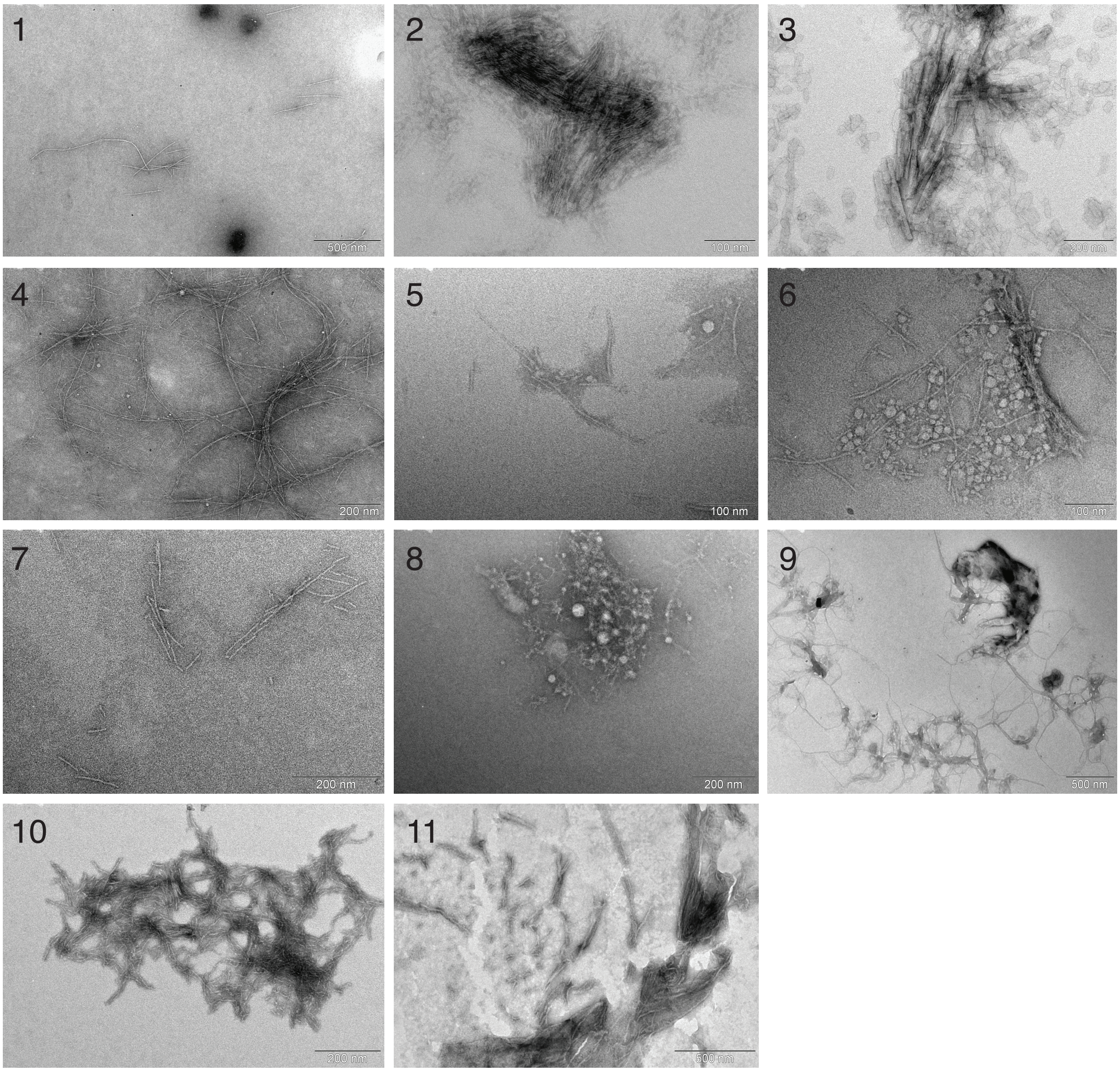

Figure S2. Morphological characterization of the peptide aggregates. Each of the 11 peptides in this study (see Table 1) were characterized by negatively stained transmission electron microscopy. The micrographs all contain fibrillar or amyloid-like structures. The same samples prepared for the FTIR measurements were used for the microscopy.

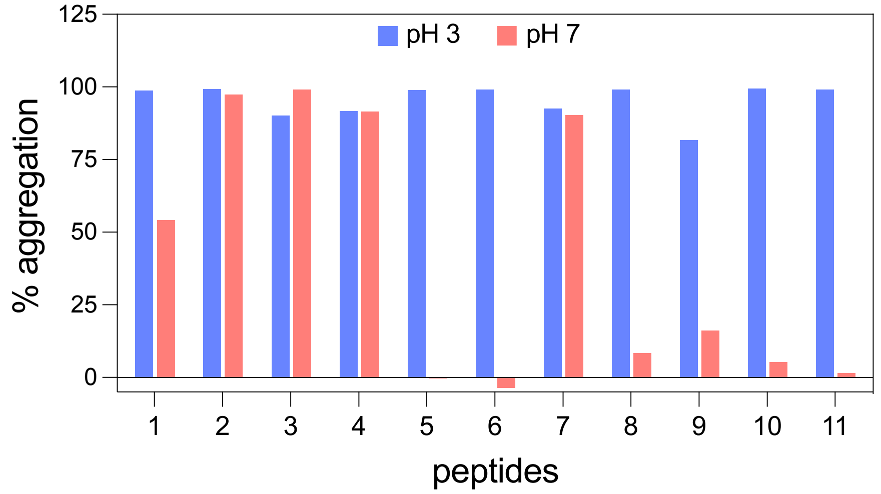

Figure S3. Peptide solubility at pH 3 and 7. Peptides used in this study (see Table 1) were aggregated at a concentration of 100 µM and the % aggregation was determined by HPLC-based supernatant analysis.

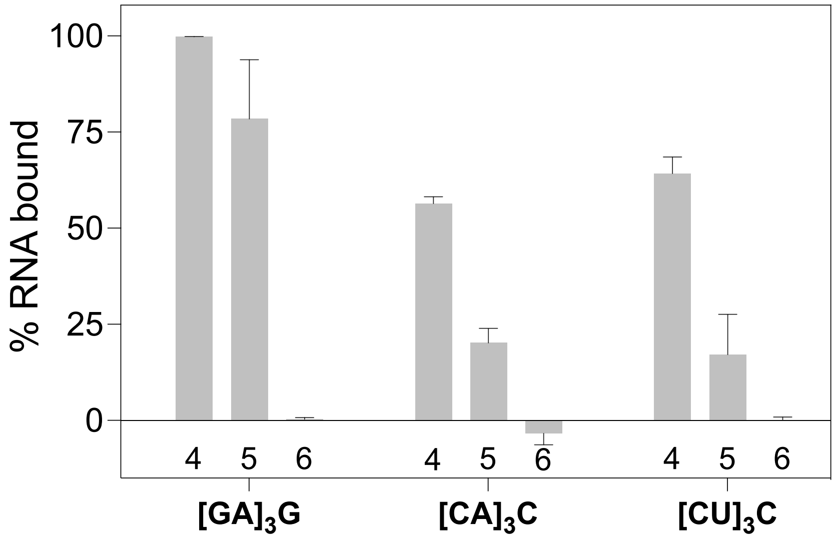

Figure S4. Charge dependence of RNA-amyloid interactions. As in Fig 2b, the requirement of a positive charge in the peptide is seen on the interaction between RNA ([GA]3G, [CA]3C and [CU]3C) and peptides **4**, **5** and **6** at pH 3. The assay was performed at room temperature in citrate-phosphate buffer with 100 µM peptide and 50 µM RNA. Errors are given as the standard deviation of two completely independent assays.

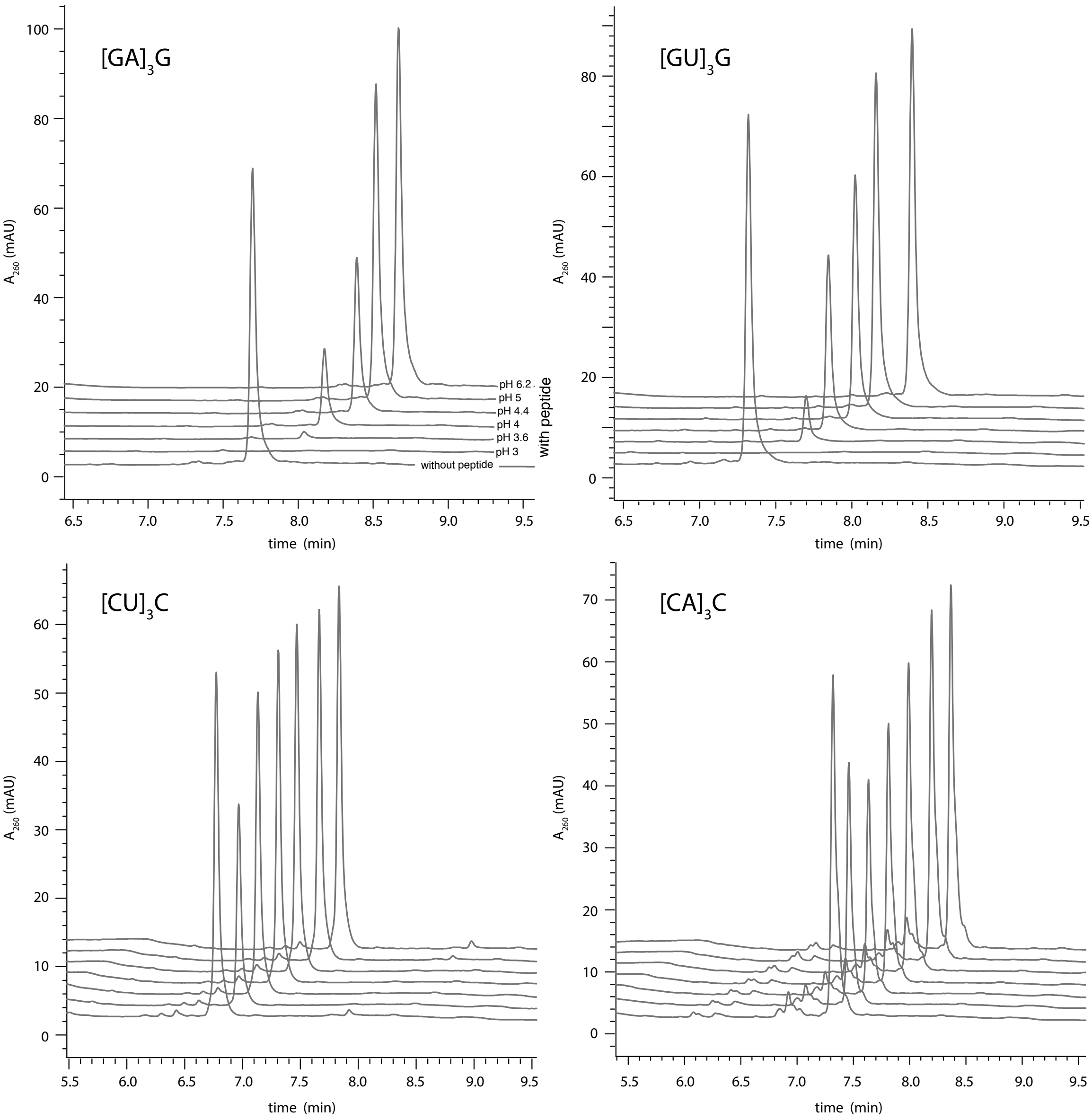

Figure S5. Quantitative estimation of the RNA-amyloid interaction by HPLC. The HPLC traces of the soluble fraction of the RNA-amyloid complex reveal the pH-dependent interaction of RNA and amyloid. The binding study was performed for **[GA]3G**, **[GU]3G**, **[CA]3C** and **[CU]3C** with peptide **7** at room temperature in citrate-phosphate buffer at different pH. The first chromatogram is for RNA only and others are for the soluble fraction in the RNA-amyloid complex at pH 3.0 – pH 6.2 (as noted in the upper left plot for **[GA]3G**). The peptide concentration was 100 µM and the RNA concentration was 50 µM. The integrated peaks areas from these plots are presented graphically as % bound RNA in Fig 2C.

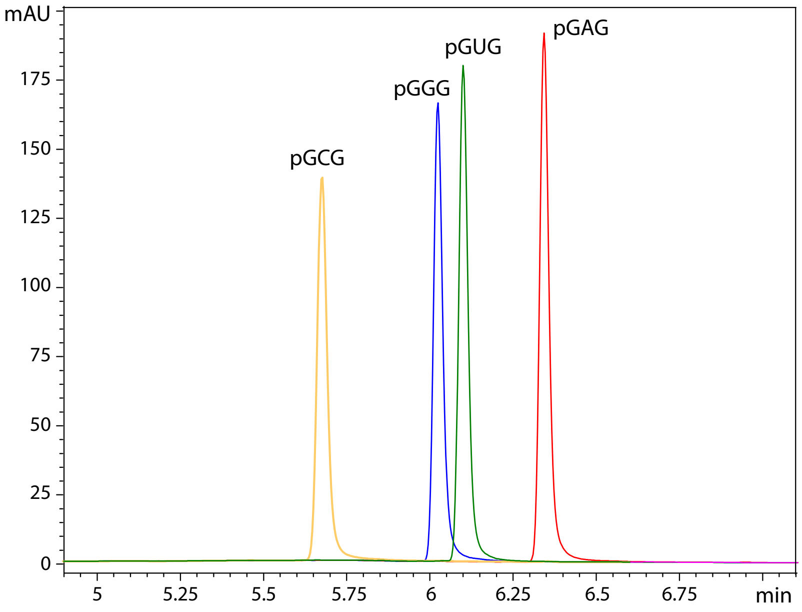

Figure S6. Mobility of pGNG RNA trinucleotides by reverse phase HPLC. The HPLC traces of the four pGNG trinucleotides are overlayed to demonstrate their elution order, which corresponds to the reverse of the order of hydrophobicity of the mononucleotide of the middle base.

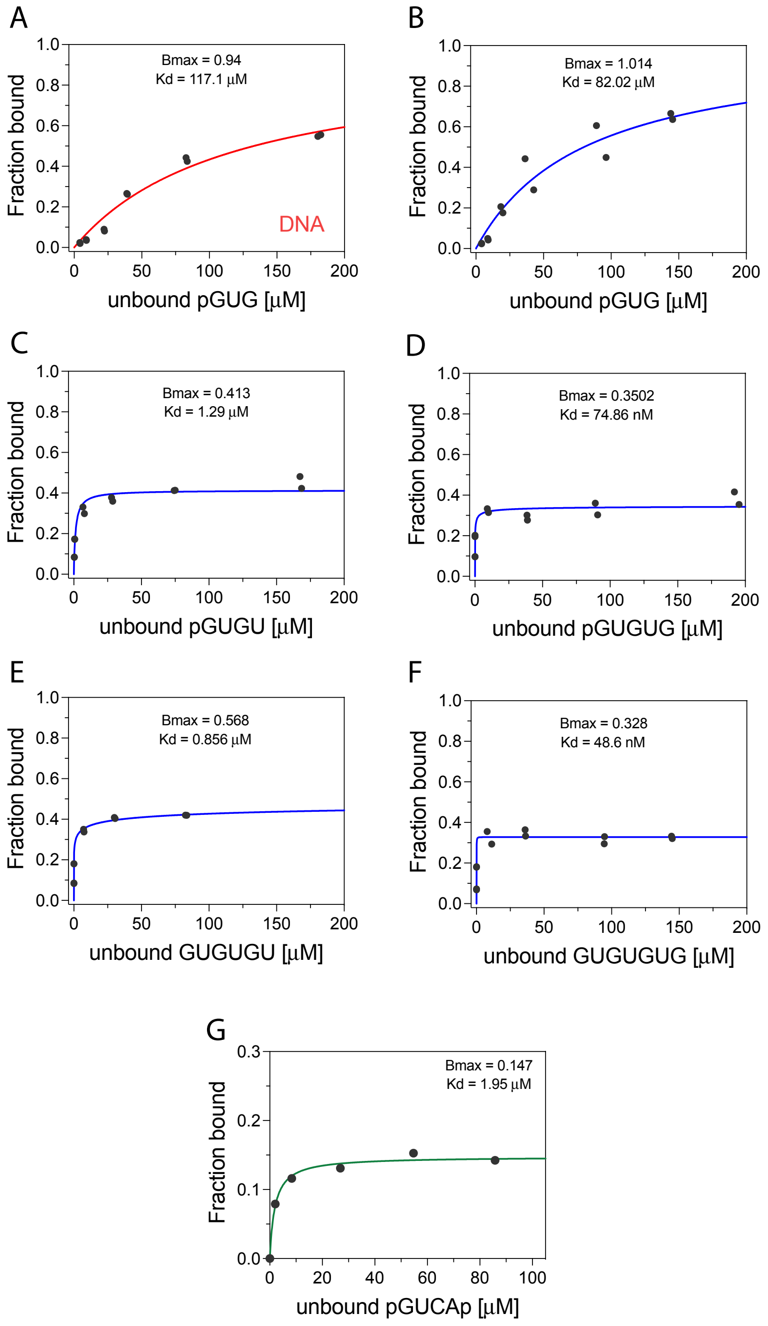

Figure S7. Affinity of the RNA-amyloid interaction. Binding dissociation constants for **pGUG, pGUGU, pGUGUG, $[GU]_3$** and **$[GU]_3G$** binding to the peptide **7** amyloid. HPLC analyses of the supernatant from mixtures of 50 μM peptide and 5-200 μM RNA were performed to obtain the fraction bound (ratio of RNA to peptide in the pellet) as a function of unbound RNA. The data were fit to a model for a single binding site to yield the K_d and maximum fraction bound. (**A**) Plot and fit of the data for pGUG (DNA backbone) binding. (**B**) the data for **pGUG** binding. (**C**) the data for **pGUGU** binding. (**D**) the data for **pGUGUG** binding. (**E**) the data for **$[GU]_3$** binding. (**F**) the data for **$[GU]_3G$** binding. (**G**) the data for **pGUCAp** binding to VAQAQINI-NH_2 peptide. The data analysis and plotting were performed with GraphPad Prism (version- 9.5.1) program.

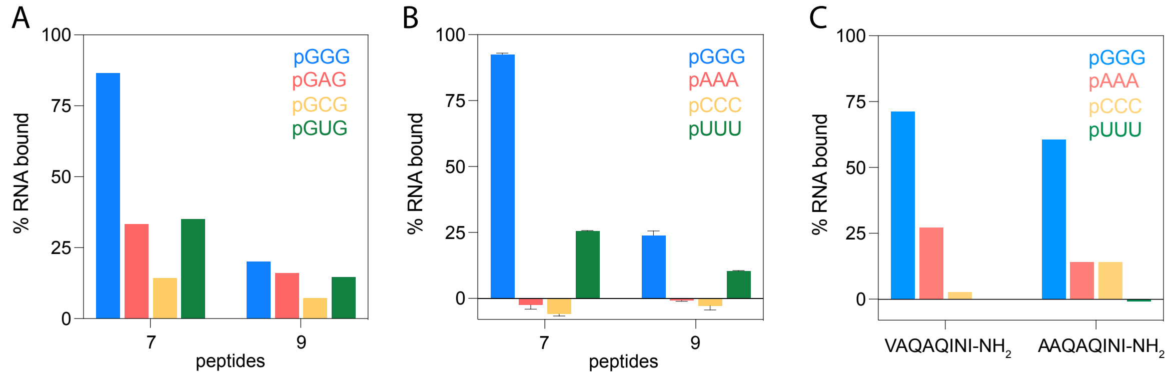

Figure S8. RNA trinucleotide binding to amyloid. All data are plotted as the %-bound RNA trinucleotide to the amyloid. (**A**) Binding of **pGGG**, **pGAG**, **pGCG** and **pGUG** with peptides **7** and **9** at pH 3. (**B**) Binding of **pGGG**, **pAAA**, **pCCC** and **pUUU** with peptides **7** and **9** at pH 3. (**C**) Binding of **pGGG**, **pAAA**, **pCCC** and **pUUU** to Val and Ala variants of peptide **S7** (**VAQAQINI-NH$_2$** and **AAQAQINI-NH$_2$**) at pH 5. The assays were performed at room temperature in citrate-phosphate buffer with 100 µM peptide (400 µM in **C**) and 25 µM RNA.

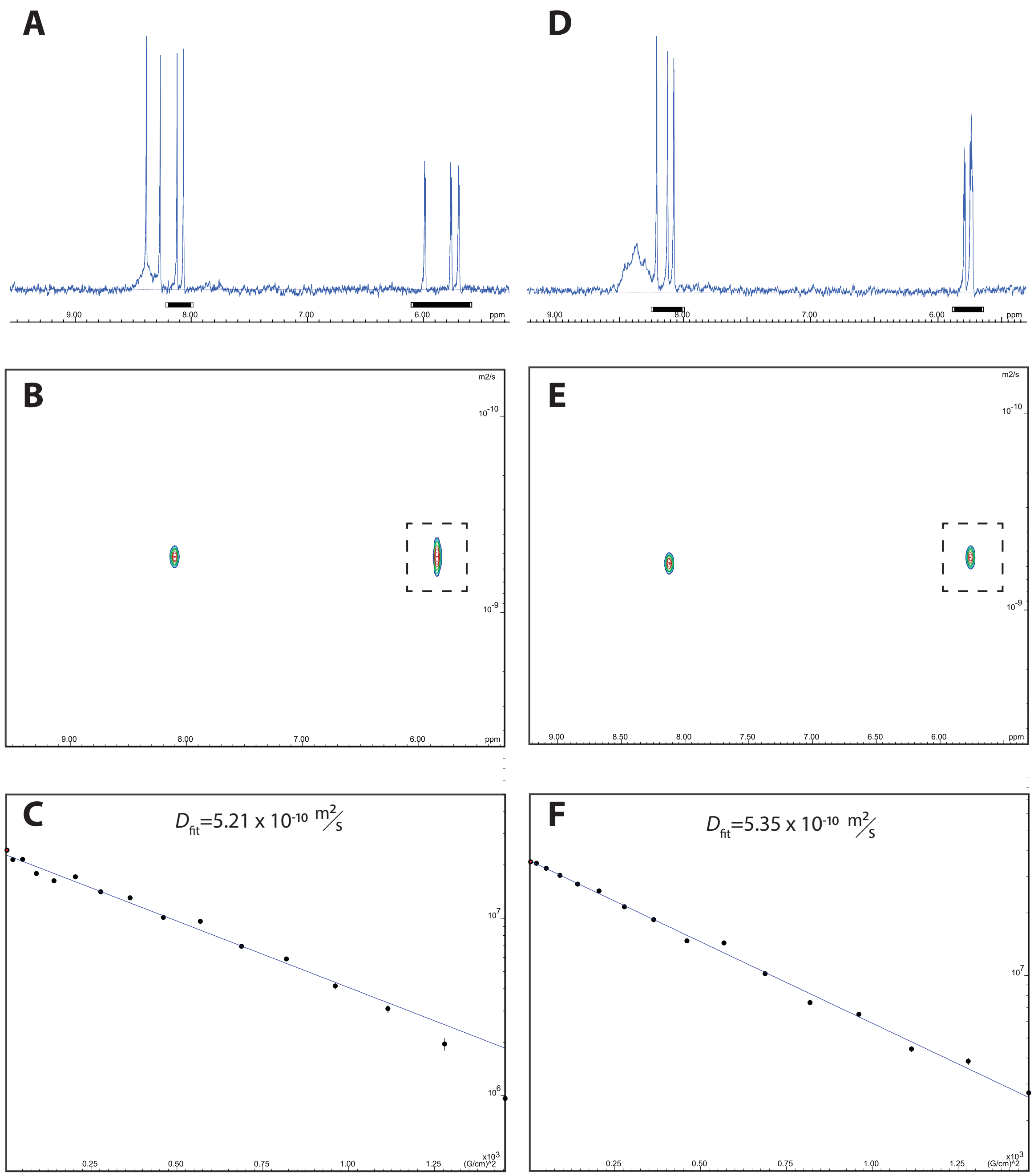

Figure S9. DOSY NMR of pGAG and pGGG

(**A**) The 1D ^{1}H spectrum of **pGAG**, (**B**) its DOSY spectrum and (**C**) the fit to the data for the peaks near 6 ppm (in the dashed-line box). (**D**) (**E**) and (**F**) are the same plots for **pGGG**.

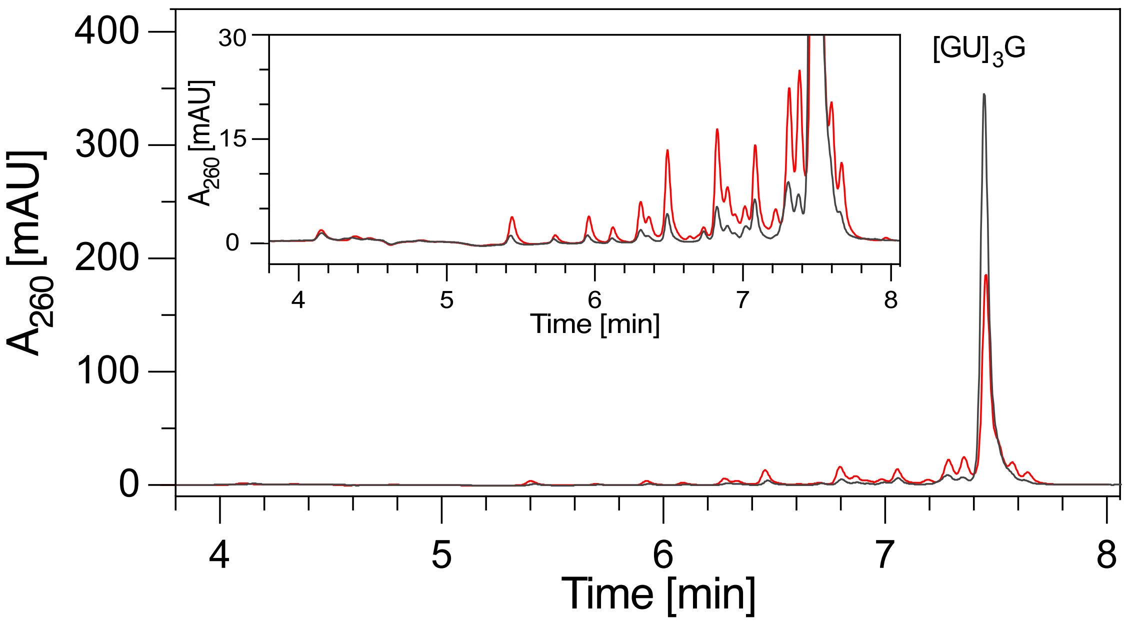

Figure S10. RNA protection by amyloid at 50 °C. HPLC chromatograms are shown for **[GU]₃G** only (red) and **[GU]₃G** in the presence of peptide **7** (black). The assay was performed by incubating RNA in the presence and absence of peptide at 50 °C for 16 h in citrate-phosphate buffer pH 2.6. The peptide concentration was 100 µM and the RNA concentration was 20 µM.

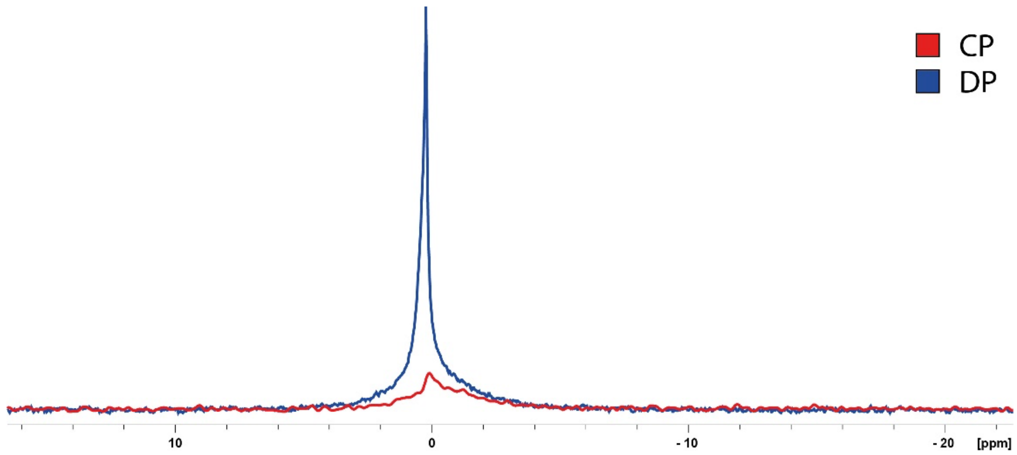

Figure S11. NMR-based evaluation of the presence of an RNA-peptide amyloid complex. 1D ^{31}P MAS NMR spectra of the peptide amyloid RNA sample IV recorded with cross polarization (CP) or direct polarization (DP) transfers. Since cross-polarization yields only signal enhancement for sufficiently immobilized species, the strong signal difference between the two spectra indicates the presence of RNA-bound to the peptide amyloid along with a large quantity of unbound RNA present in the supernatant in the NMR rotor.

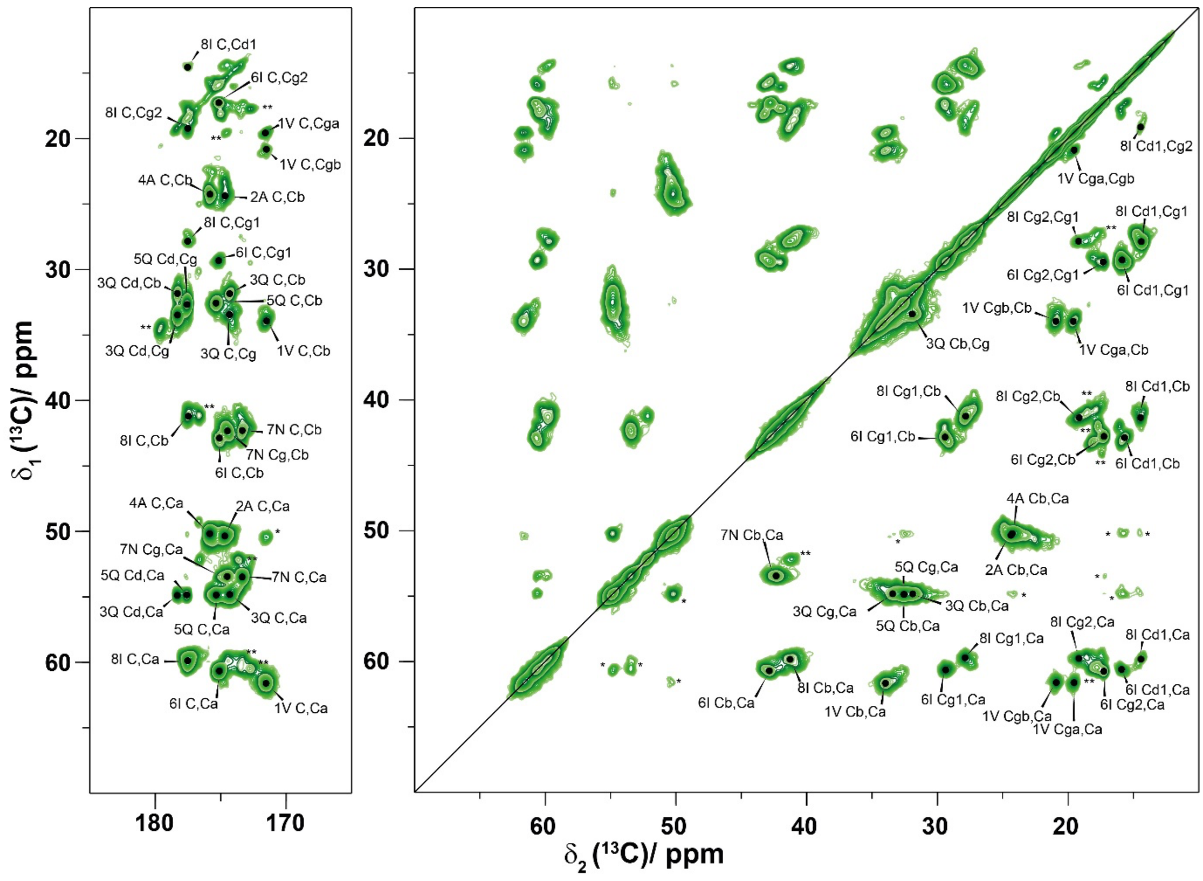

Figure S12. Solid state NMR sequential assignment of the peptide amyloid. Part of the 2D [^{13}C,^{13}C] 20 ms DARR Spectrum of sample I is shown with the sequential assignment employing one letter amino acid code indicated.

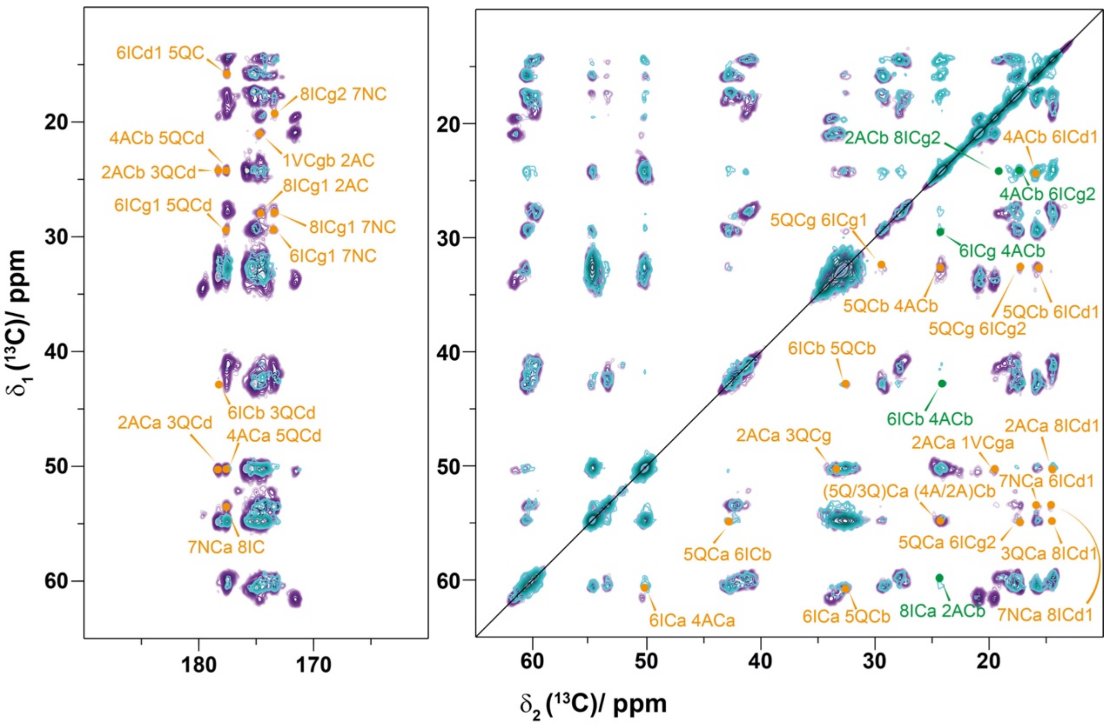

Figure S13. Distance restraint collection of the peptide amyloid. 2D [^{13}C,^{13}C]-DARR (with 150 ms mixing time) spectrum of the peptide amyloid in purple superimposed with a corresponding 2D [^{13}C,^{13}C]-CHHC (400 μs) spectrum in cyan yields sequential and long range distance restraints as indicated in yellow for cross peaks observed in the 2D [^{13}C,^{13}C]-DARR spectrum and labeled in green observed in the 2D [^{13}C,^{13}C]-CHHC spectrum.

A

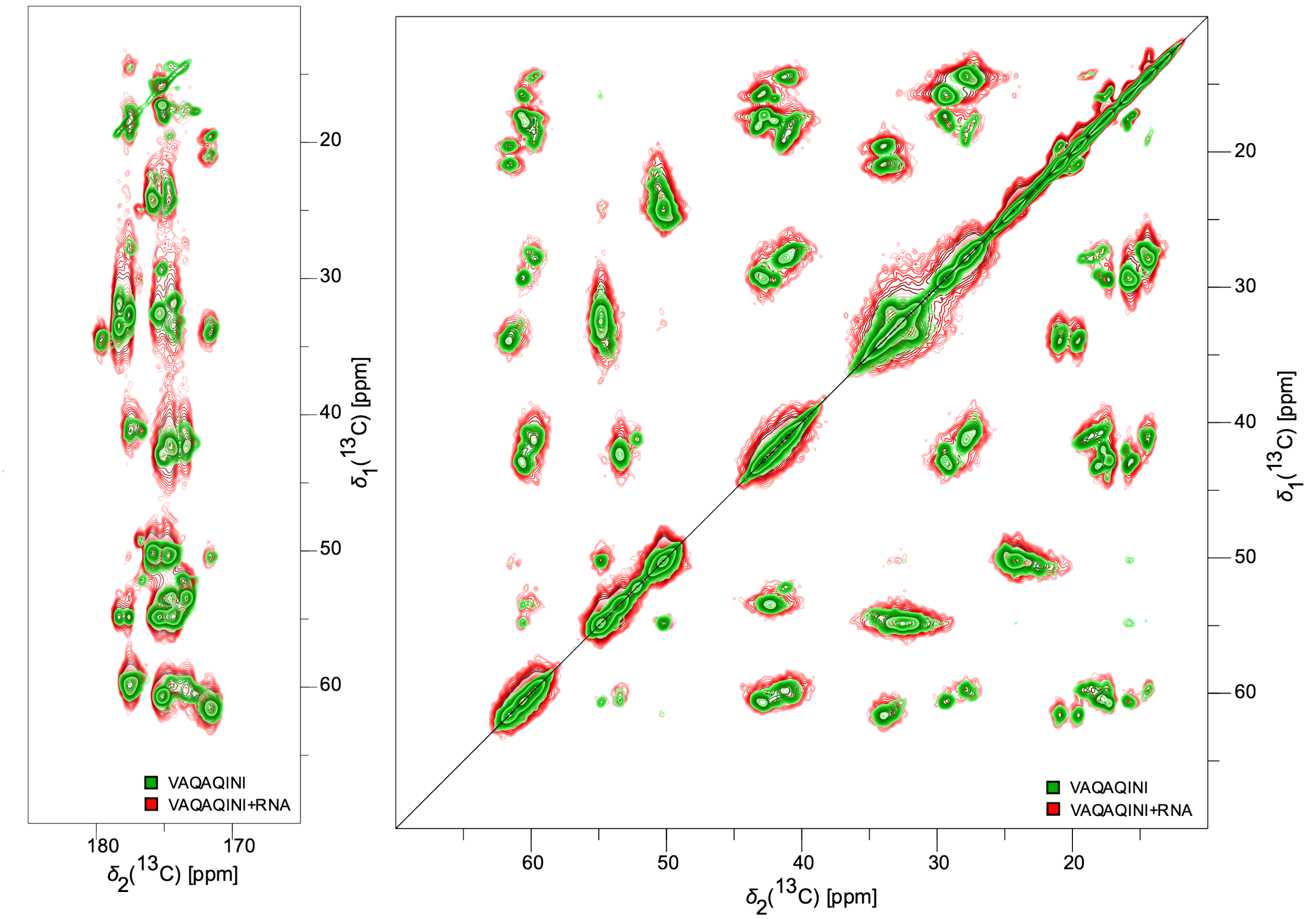

B

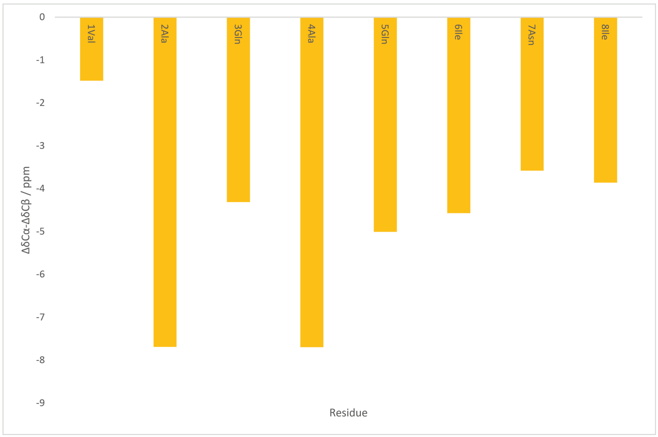

Figure S14. (A) Superposition between 2D [^{13}C,^{13}C] DARR spectrum of the peptide amyloid (in green) with the corresponding spectrum of the peptide amyloid – RNA complex (in red) indicates the same structure of the peptide amyloid in absence or in presence of RNA. Broader resonances in the red spectrum might be related to a lower static magnetic field used. **(B)** Solid-state NMR-derived secondary chemical-shift differences as a function of residue number for the VAQAQINI-NH_2 amyloid.

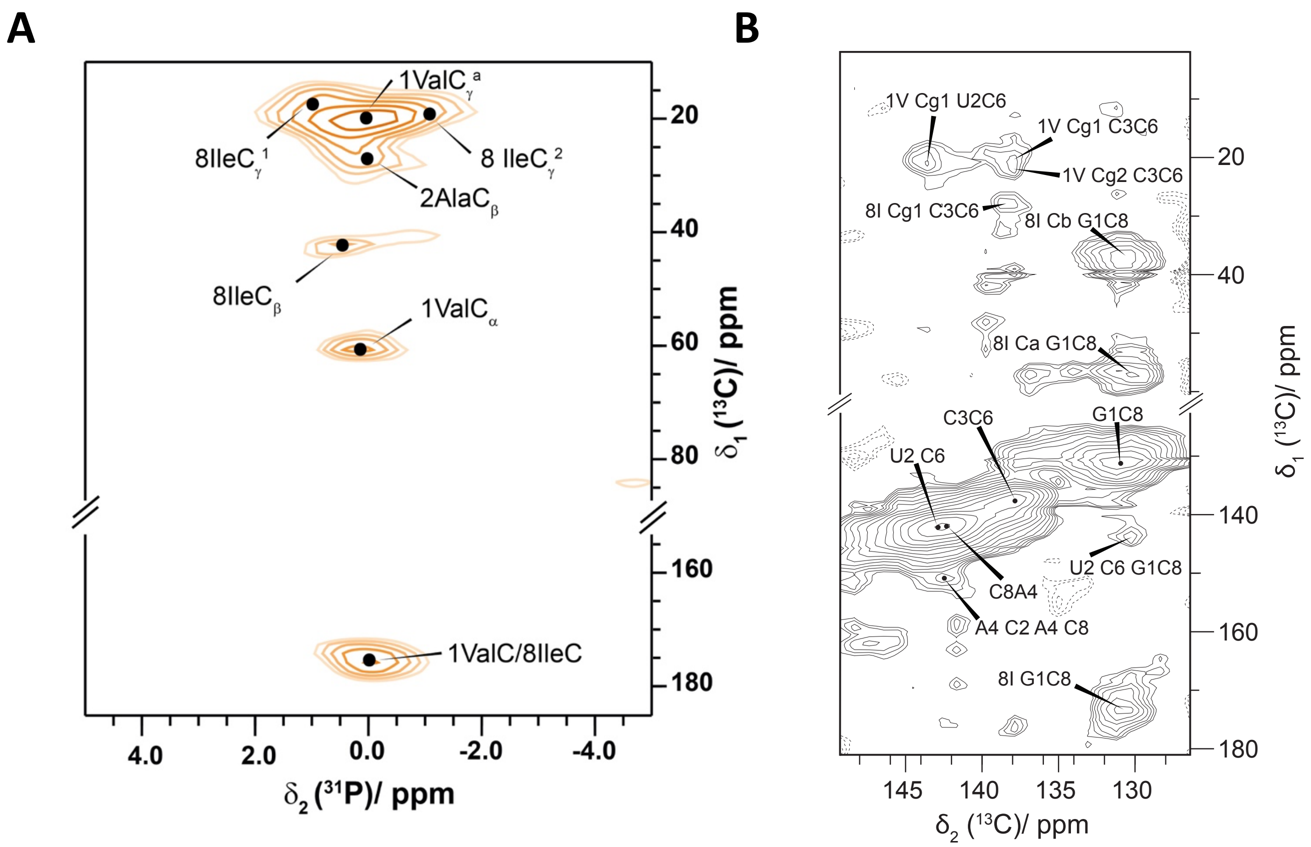

Figure S15. Distance restraint collection between RNA and peptide. (A) [^{13}C,^{31}P] CHHP spectrum of the peptide amyloid RNA complex with sample IV (resonances were assigned as described in the text) and **(B)** [^{13}C,^{13}C] 450 ms DARR spectrum with sample V. Cross peaks are labeled with one letter code.

Suppl. Fig. S16 (page 1 of 9)

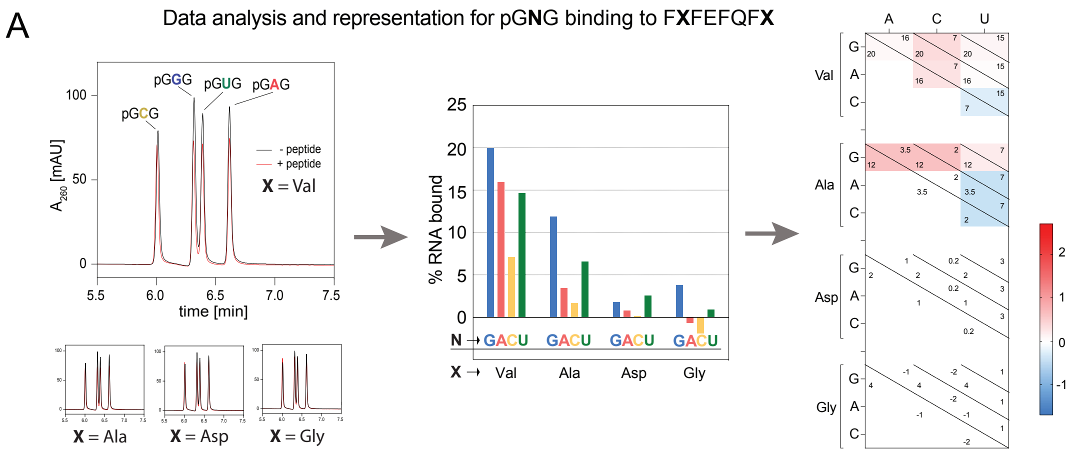

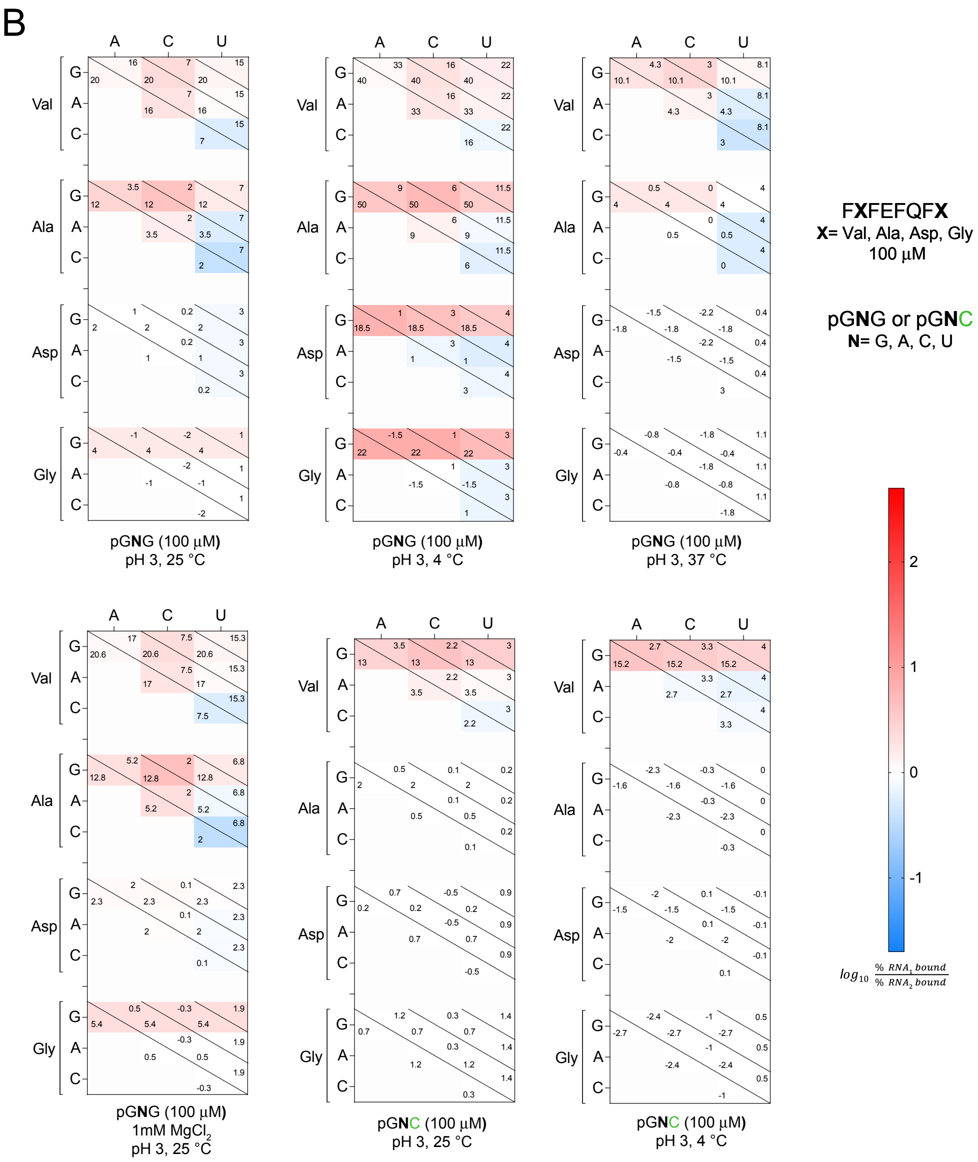

Suppl. Fig. S16 (page 2 of 9)

C

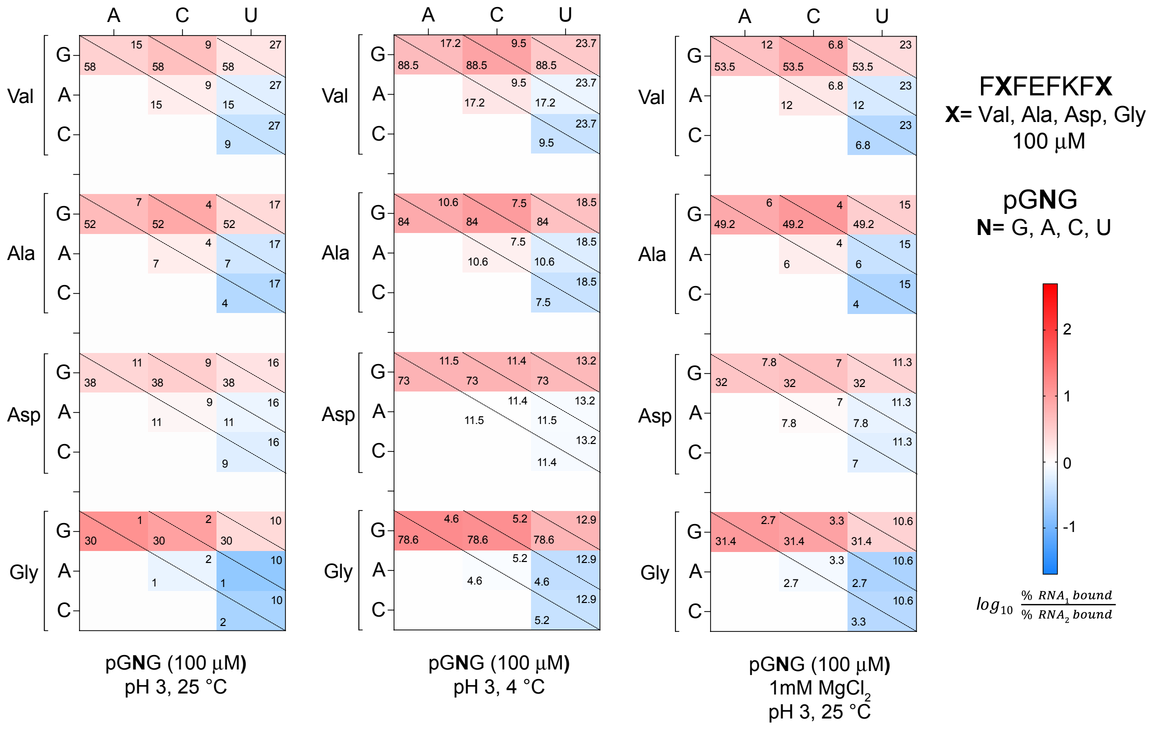

D

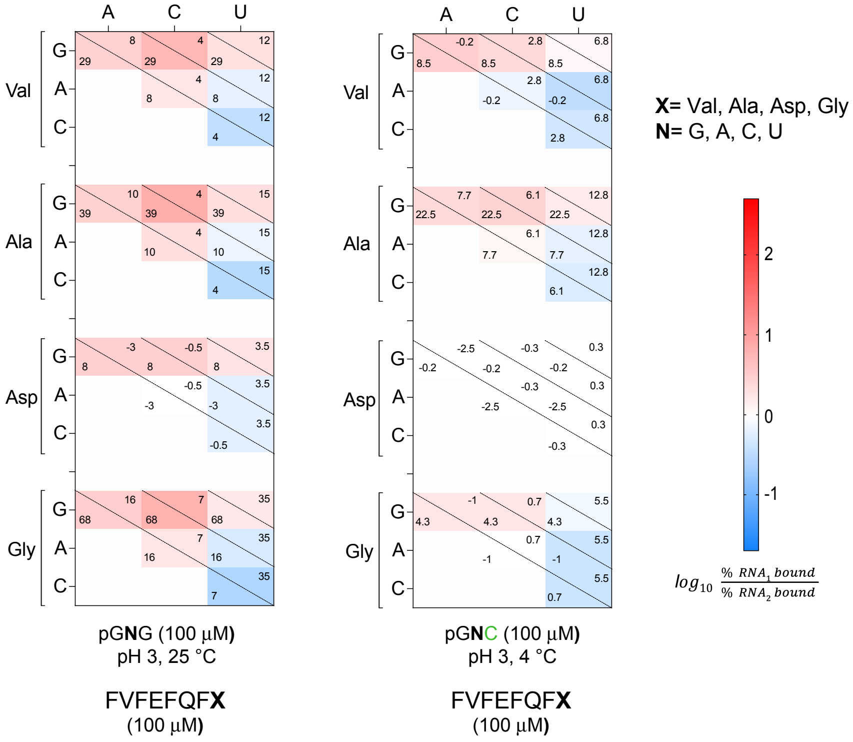

Suppl. Fig. S16 (page 3 of 9)

E

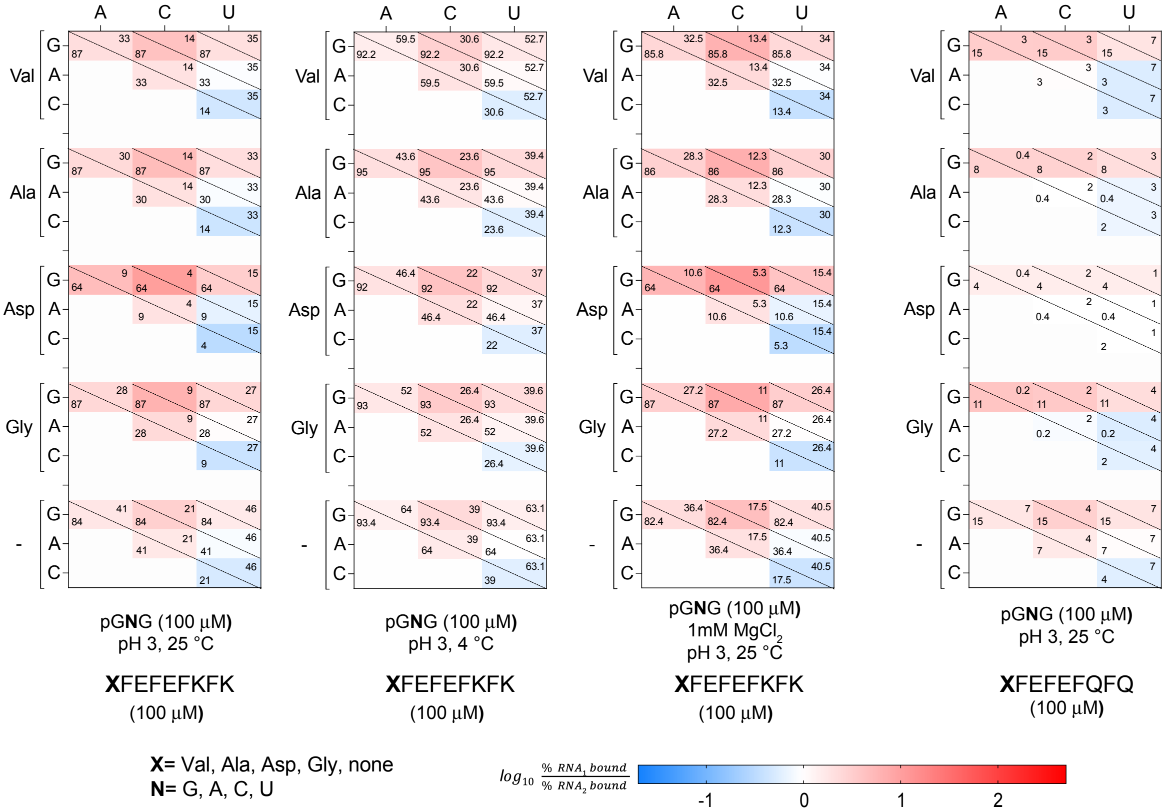

F

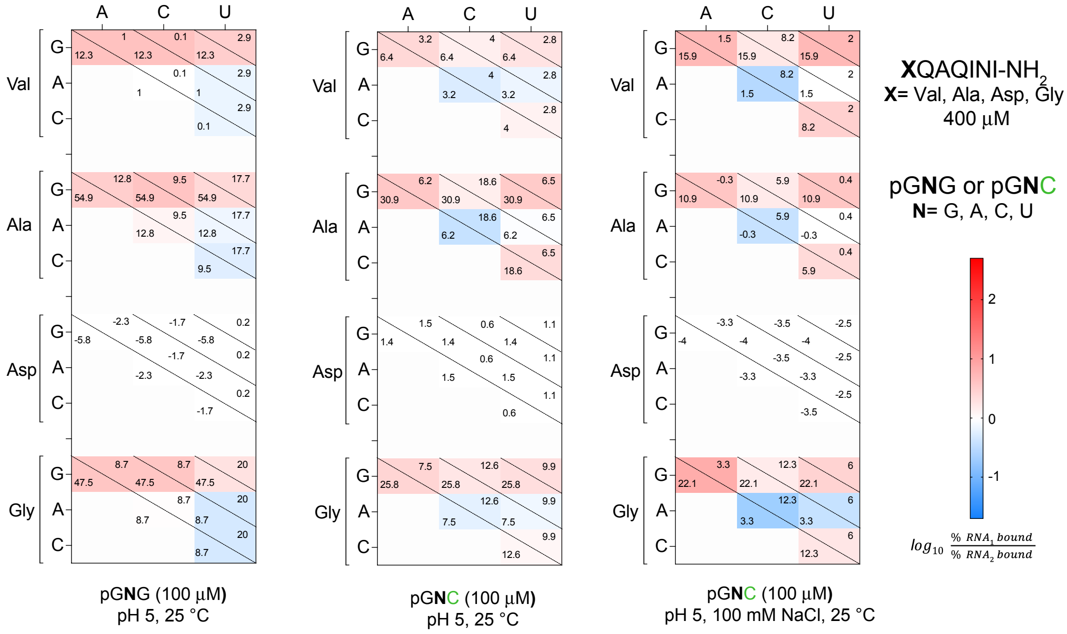

Suppl. Fig. S16 (page 4 of 9)

G

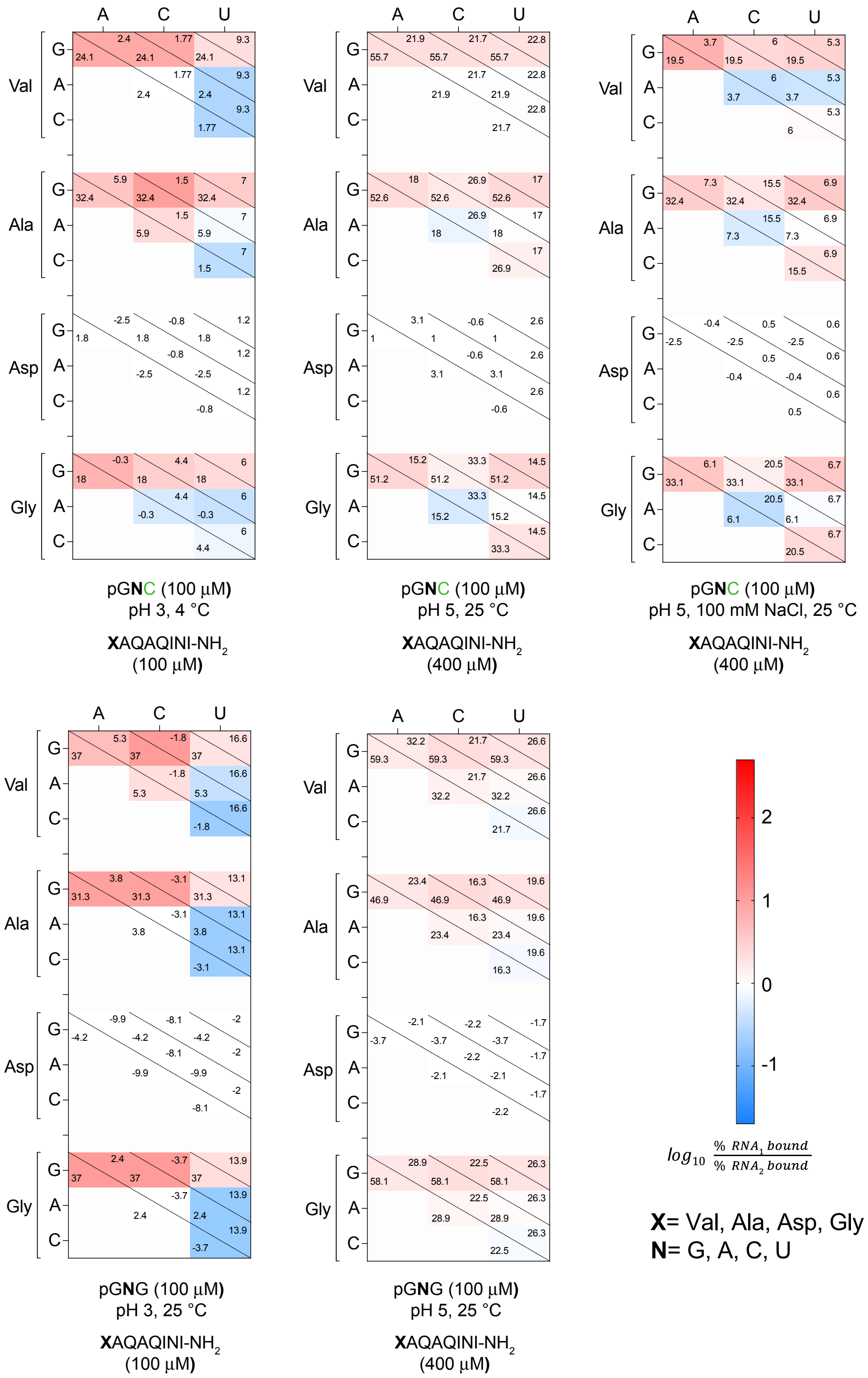

H

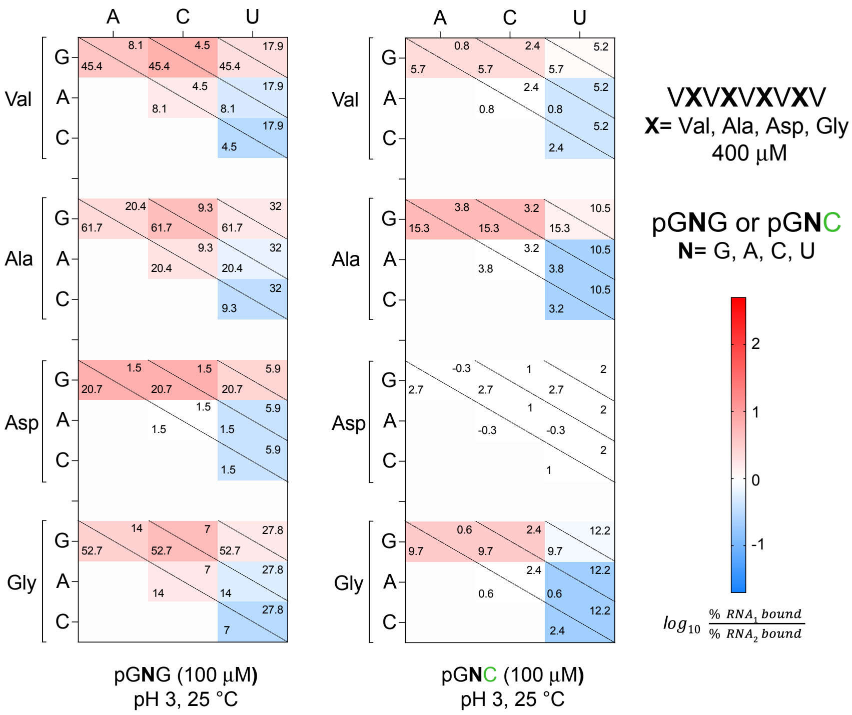
A C U
Val
Ala
Asp
Gly
G
A
C
VXVXVXVXV
X= Val, Ala, Asp, Gly
400 μM
pGNG or pGNC
N= G, A, C, U
2
1
0
-1
$log_{10} \frac{\% RNA_1 bound}{\% RNA_2 bound}$
pGNG (100 μM)
pH 3, 25 °C
pGNC (100 μM)
pH 3, 25 °C

I

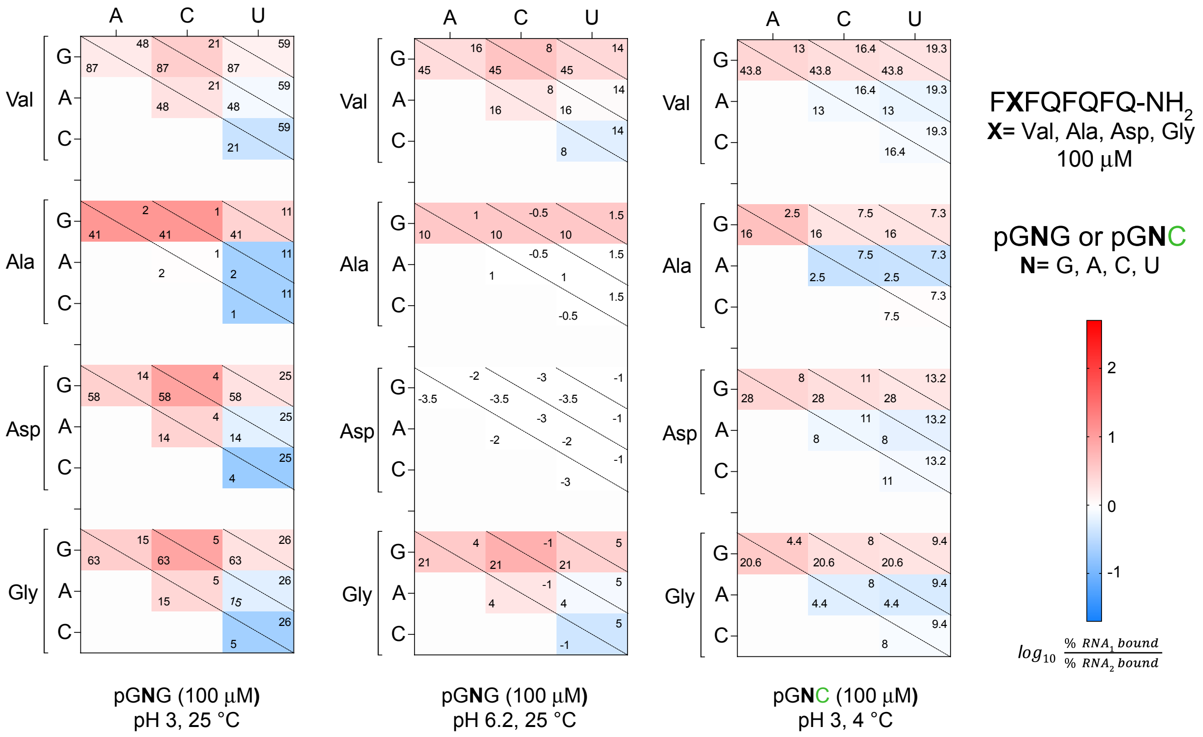
A C U
Val
Ala
Asp
Gly
G
A
C
FXFQFQFQ-NH_2
X= Val, Ala, Asp, Gly
100 μM
pGNG or pGNC
N= G, A, C, U
2
1
0
-1
$log_{10} \frac{\% RNA_1 bound}{\% RNA_2 bound}$
pGNG (100 μM)
pH 3, 25 °C
pGNG (100 μM)
pH 6.2, 25 °C
pGNC (100 μM)
pH 3, 4 °C

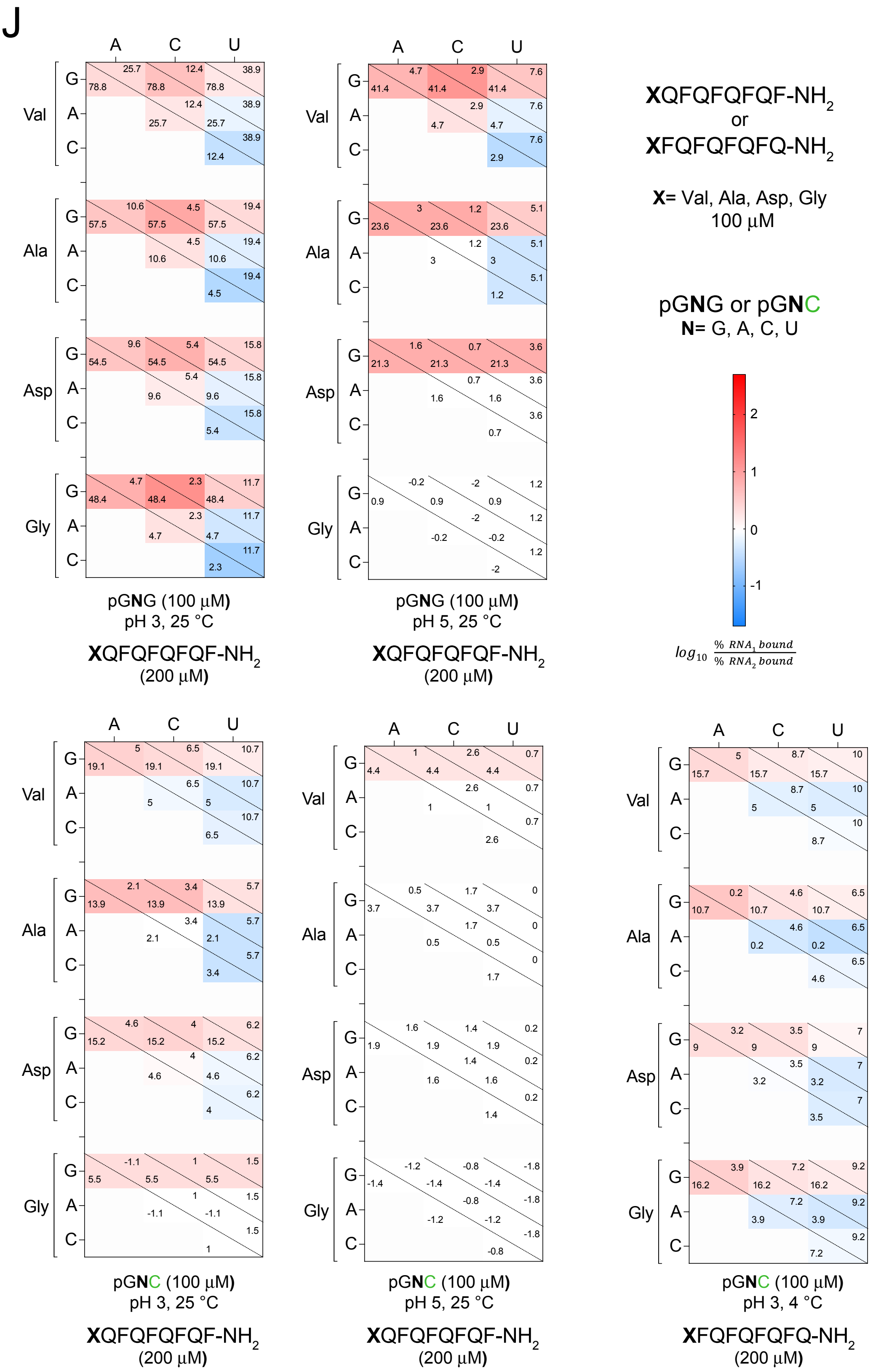
J
XQFQFQFQF-NH2
or
XFQFQFQFQ-NH2
X= Val, Ala, Asp, Gly
100 µM
pGNG or pGNC
N= G, A, C, U
pGNG (100 µM)
pH 3, 25 °C
XQFQFQFQF-NH2
(200 µM)
pGNG (100 µM)
pH 5, 25 °C
XQFQFQFQF-NH2
(200 µM)
pGNC (100 µM)
pH 3, 25 °C
XQFQFQFQF-NH2
(200 µM)
pGNC (100 µM)
pH 5, 25 °C
XQFQFQFQF-NH2
(200 µM)
pGNC (100 µM)
pH 3, 4 °C
XFQFQFQFQ-NH2
(200 µM)

Suppl. Fig. S16 (page 7 of 9)

K

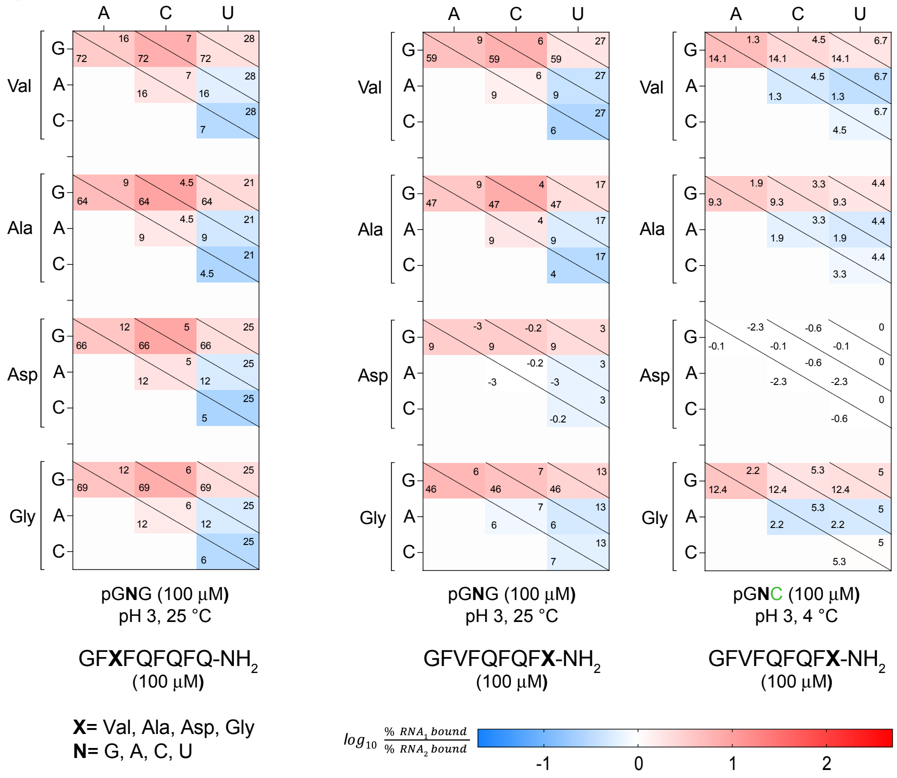

L

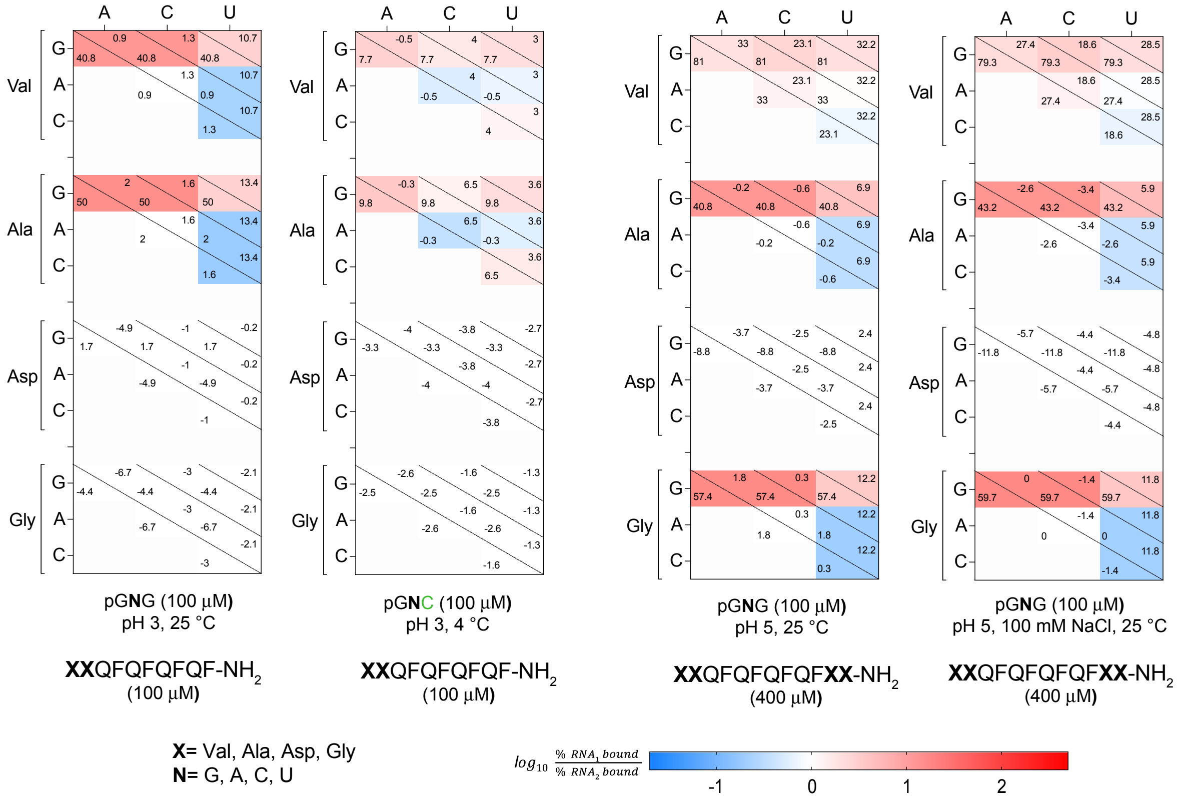

Suppl. Fig. S16 (page 8 of 9)

M

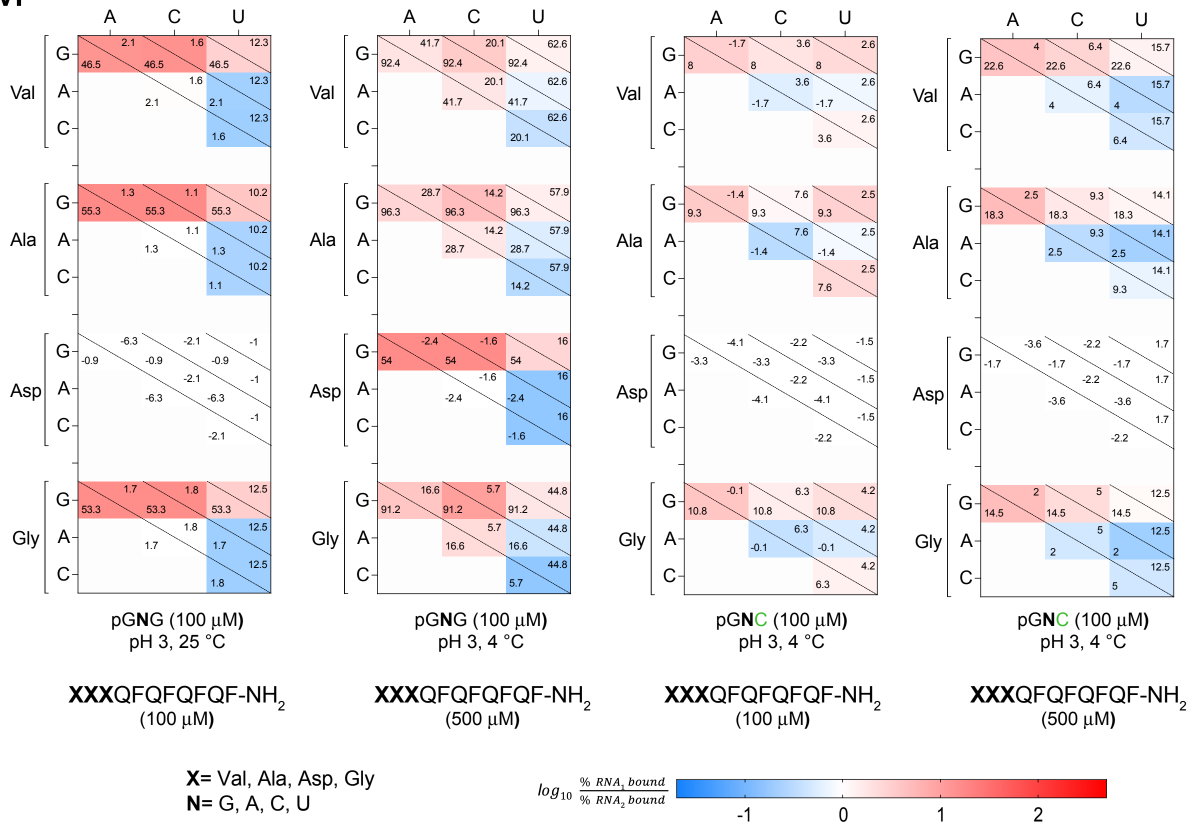

N

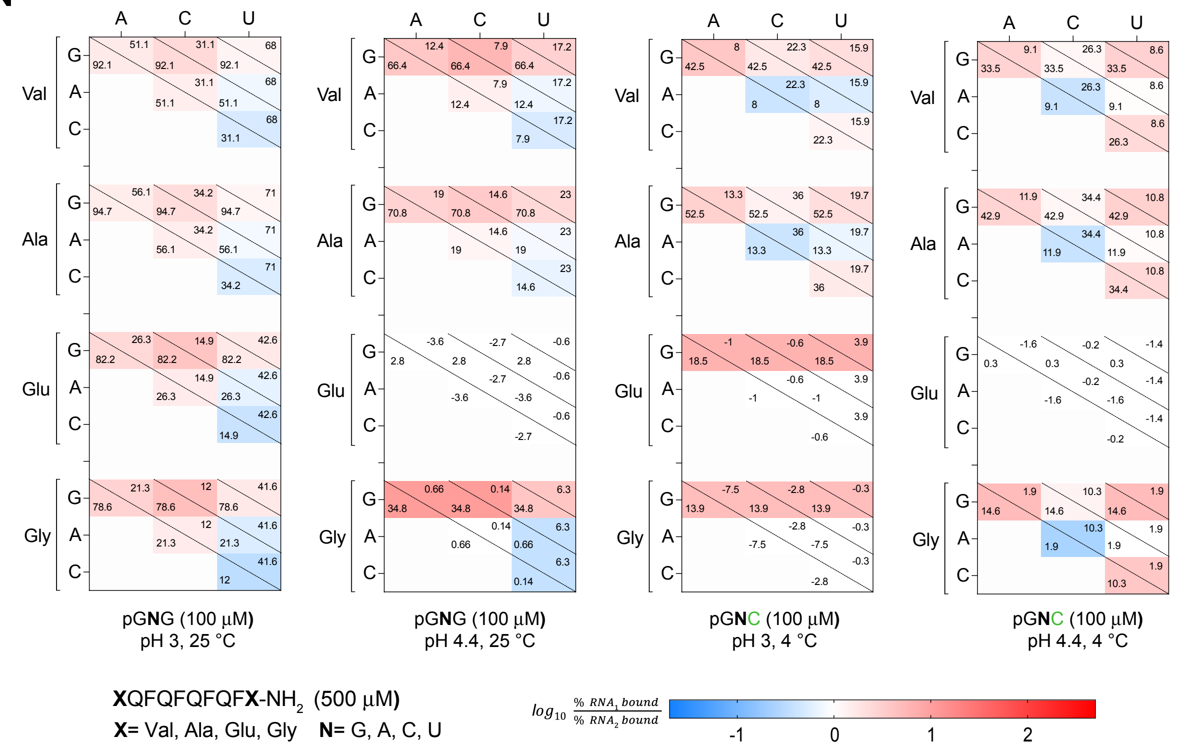

O

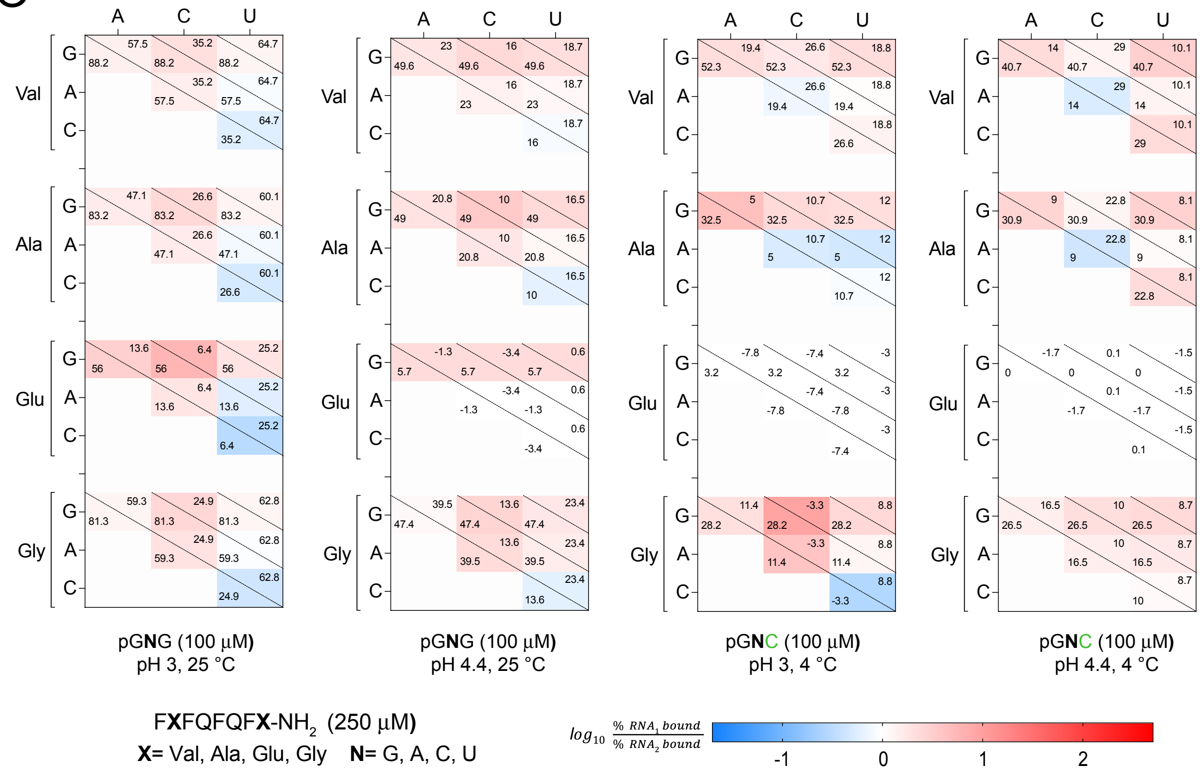

Figure S16. Sequence-selective RNA-amyloid interactions. (A) Systematic data interpretation steps of the RNA trinucleotide-amyloid interactions. In the example, the HPLC chromatograms (left) are used to calculate the % RNA bound to peptide amyloid FXFEFQFX (X= Val, Ala, Asp, Gly) for each of the four pGNG (N= G, A, C, U) trinucleotides (middle). To visualize the relative binding between all 6 pairs of RNA trinucleotides to a particular peptide, the log of the ratio of % bound RNA for each pair is displayed as a heatmap (right) with only the variable nucleotide displayed on the axes of heatmap plots. To minimize the bias from errors, all measured values of less than 4% are set to 2% for the log-ratio calculation, because for these weak binders, small errors are large relative to the measured values. For reference, the measured absolute %-bound is given by numbers on each side of the diagonal line. (B)-(O) The log ratio of %-bound RNA to a particular peptide for each of the pGNG or pGNC trinucleotide sets is plotted for a selection of the tested peptide sets (Table S1) demonstrating the variable specificity in the amyloid-RNA interactions. Each panel presents results from one (or two related) peptide sets at different assay conditions. Peptide and RNA sequences and the concentrations used are noted on each plot as are the assay conditions which include variations in temperature, pH and salt.

Table S1. Amyloidogenic peptide sets used in the sequence-selective RNA trinucleotide-amyloid interaction study

Set	Composition [a]	Ionization at neutral pH [b]					
		N	Sidechain [c]	Sidechain [d]	C	Sum [c]	Sum [d]
**S1**	F**X**FEFQF**X**	+	-	---	-	-1	-3
**S2**	F**X**FEFKF**X**	+	-+	--+-	-	0	-2
**S3**	FVFEFQF**X**	+	-	--	-	-1	-2
**S4**	**X**FEFEFKFK	+	--++	---++	-	0	-1
**S5**	**X**FEFEFQFQ	+	--	---	-	-2	-3
**S6**	**X**QAQINI-NH_2	+		-		+1	0
**S7**	**X**AQAQINI-NH_2	+		-		+1	0
**S8**	V**X**V**X**V**X**V**X**V	+		----	-	0	-4
**S9**	F**X**FQFQFQ-NH_2	+		-		+1	0
**S10**	**X**QFQFQFQF-NH_2	+		-		+1	0
**S11**	**X**FQFQFQFQ-NH_2	+		-		+1	0
**S12**	GF**X**FQFQFQ-NH_2	+		-		+1	0
**S13**	GFVFQFQF**X**-NH_2	+		-		+1	0
**S14**	**XX**QFQFQFQF-NH_2	+		--		+1	-1
**S15**	**XX**QFQFQFQF**XX**-NH_2	+		----		+1	-3
**S16**	**XXX**QFQFQFQF-NH_2	+		---		+1	-2
**S17***	**X**QFQFQFQF**X**-NH_2	+		--		+1	-1
**S18***	F**X**FQFQF**X**-NH_2	+		--		+1	-1

[a]Amino acid residues are represented in the standard single letter code; NH_2 is for amidated C-terminus; **X** = G, A, V or D (*or E in S17 and S18)
[b] Dominant ionization states of the ionizable groups of the peptide at neutral pH are listed as the charges on the N-terminus, sidechains and C-terminus. The listed ionization states are based on individual pKas of the groups and are expected to vary depending on buffer pH and the aggregation state of the peptide.
[c] Side chains with X = G, A and V
[d] Side chains with X = D or E

Table S2. Restraint and structure statistics

Quantity	
Conformational restraints (per monomer):[a]	
Intra-amyloid distance restraints[a]	49
Inter-sheet distance restraints[b]	6
RNA-peptide distance restraints[c]	30
Restrained hydrogen bonds[d]	7
Dihedral angle restraints (ϕ/ψ)	12
Restraint violations:[e]	
CYANA target function value (Å^2)	6.59 ± 0.21
RMS distance restraint violation (Å)	0.029 ± 0.001
Maximal distance restraint violation (º)	0.38± 0.07
RMS dihedral angle restraint violation (Å)	1.93 ± 0.08
Maximal dihedral angle restraint violation (º)	8.06 ± 0.77
RMSD to mean for the central 2 peptide monomers:	
Backbone of residues (Å)	0.1 ± 0.03
All heavy atoms of residues (Å)	0.79 ± 0.1
RMSD to mean for the RNA - peptide amyloid complex	
All heavy atoms (Å)	1.5 ± 0.4

[a]Each group of symmetrically equivalent distance restraints is counted as a single restraint. Distance restraints with multiple assignments are classified by the assignment spanning the shortest residue range.
[b]Each group of symmetrically equivalent distance restraints is counted as a single restraint. Distance restraints with multiple assignments are classified by the assignment spanning the shortest residue range. The distance restraints include one artificial distance restraint helping to find a good energy minimum (see text).
[c]Each group of symmetrically equivalent distance restraints is counted as a single restraint. Distance restraints with multiple assignments are classified by the assignment spanning the shortest residue range. The distance restraints include one additional distance restraint derived indirectly from interaction studies using RNA variants (see text).
[d]Each hydrogen bond was restrained by two upper and two lower distance bounds.
[e]Where applicable, the average value and the standard deviation over the 10 conformers that represent the NMR structure are given.

Table S3. Parameters used for solid-state NMR experiments

Experiment	DARR 20 ms (Sample I)	DARR 150 ms (Sample I)	NCA (Sample I)	NCO (Sample I)	DARR 20 ms (Sample II)	DARR 150 ms (Sample II)	PAR 8 ms (Sample III)
MAS frequency [kHz]	17	17	17	17	17	17	17
**pulse ^{1}H power/W – 100 kHz**	110	93.5	92	92	82	82	470
**pulse ^{13}C power/W – 50 kHz**	140	100	100	100	77	77	245
**pulse ^{15}N power/W – 50 kHz**	-	-	275	275	-	-	-
**Transfer 1**	HC-CP	HC-CP	HN-CP	HN-CP	HC-CP	HC-CP	HC-CP
field [kHz] -^{1}H	60	60	60	60	60	60	60
field [kHz] -X	44.3	70.7	46.7	46.7	78.5	78.5	46.3
shape	tangent	tangent	tangent	tangent	tangent	tangent	tangent
carrier [ppm]	-	-	-	-	-	-	
time [ms]	0.8	0.9	1.3	1.3	1.2	1.2	0.9
**Transfer 2**	DARR	DARR	NC-CP	NC-CP	DARR	DARR	PAR
field [kHz] -^{1}H	17	17	90	90	17	17	45
field [kHz] -^{13}C	-	-	6	6	-	-	39.8
field [kHz] - ^{15}N	-	-	9.9	9.9	-	-	-
shape	-	-	tangent	tangent	-	-	-
carrier [ppm]	-	-	CA	CO	-	-	-
time [ms]	20	50	8	8	20	150	8
t_1 increments	2560	2560	1792	1792	2560	2560	3072
sweep width (t_1) [kHz]	81.758	100	66.667	66.667	100	100	100
max. acq time (t_1) [ms]	15.66	12.80	13.44	13.44	12.8	12.80	15.36
t_2 increments	3072	3072	2304	2304	3072	3072	1792
sweep width (t_2) [kHz]	100	100	100	100	100	100	164.127
max. acq time (t_2) [ms]	15.36	15.36	11.52	11.52	15.36	15.36	13.972
^{1}H Spinal64 Decoupling power [kHz]	90	90	90	90	90	90	90
interscan delay [s]	2.7	2.7	2	2	2.7	2.7	2.7
number of scans	12	12	8	8	12	12	88

Table S4. Parameters used for solid-state NMR experiments

Experiment	PDSD (Sample III)	DARR 20 ms (Sample III)	DARR 20 ms (Sample IV)	NCA (Sample IV)	NCO (Sample IV)	DARR 150 ms and 450 ms (Sample V)
MAS frequency [kHz]	17	17	17	17	17	17
**pulse ^{1}H power/W – 100 kHz**	470	110	119	230	230	106.2
**pulse ^{13}C power/W – 50 kHz**	245	150	43	185	133	89.1
**pulse ^{15}N power/W – 50 kHz**	-	-	-	210	233	-
**Transfer 1**	HC-CP	HC-CP	HC-CP	HN-CP	HN-CP	HC-CP
field [kHz] -^{1}H	60	60	60	60	60	60
field [kHz] -X	33.5	42.8	50	49	49.5	74.9
shape	tangent	tangent	tangent	tangent	tangent	tangent
carrier [ppm]	-	-	-	-	-	-
time [ms]	0.8	0.9	1	1.5	1.5	1.2
**Transfer 2**	C-C mixing	DARR	DARR	NC-CP	NC-CP	DARR
field [kHz] -^{1}H	-	-	17	90	90	17
field [kHz] -^{13}C	-	-	-	6	6	-
field [kHz] - ^{15}N	-	-	-	10	22.3	-
shape	-	-	-	tangent	-	-
carrier [ppm]	-	-	-	CA	CO	-
time [ms]	5	20	20	5	5	150
t_1 increments	-	2560	2048	3072	3072	600
sweep width (t_1) [kHz]	-	100	100	100	100	100
max. acq time (t_1) [ms]	-	12.8	10.24	15.36	15.36	3
t_2 increments	1024	3072	2048	1536	1536	3072
sweep width (t_2) [kHz]	100	100	100	66.667	66.667	100
max. acq time (t_2) [ms]	5.12	15.36	10.24	11.52	11.52	15.36
^{1}H Spinal64 Decoupling power [kHz]	90	90	90	90	90	90
interscan delay [s]	1.5	2.7	2	3	3	2.7
number of scans	4	8	64	4	8	16

Table S5. Parameters used for solid-state NMR experiments

**Experiment**	**CHHC (Sample I)**	**CHHC (Sample II)**	**PAIN 6 ms (Sample III)**	**CHHP (Sample IV)**	**^{31}P PDSD (Sample IV)**	**^{31}P DP (Sample IV)**
MAS frequency [kHz]	17	17	17	17	17	17
**pulse ^{1}H power/W – 100 kHz**	93.5	82	470	80	80	100
**pulse ^{13}C power/W – 50 kHz**	100	77	245	83	-	-
**pulse ^{31}P power/W – 50 kHz**	-	-	445	79	79	40
**Transfer 1**	HC-CP	HC-CP	HN-CP	HC-CP	HP-CP	DP
field [kHz] -^{1}H	60	60	60	60	60	-
field [kHz] -X	70.7	78.5	45.3	43.2	35.6	40
shape	tangent	tangent	tangent	tangent	tangent	-
carrier [ppm]	-	-	-	-	-	-
time [ms]	0.9	1.2	0.9	1.25	3.5	-
**Transfer 2**	CH-CP	CH-CP	PAIN	CH-CP	C-C mixing	-
field [kHz] -^{1}H	60	60	40.4	60	-	-
field [kHz] -^{13}C	70.7	78.5	39.1	43.2	-	-
field [kHz] – ^{15}N	-	-	37.5	-	-	-
shape	tangent	tangent		tangent	-	-
carrier [ppm]		-	-	-	-	-
time [ms]	0.9	1.2	6	1.25	5	-
**Transfer 3**	H-H mixing	H-H mixing	-	H-H mixing	-	-
field [kHz] -^{1}H	-	-	-	-	-	-
field [kHz] -^{13}C	-	-	-	-	-	-
field [kHz] - ^{15}N	-	-	-	-	-	-
shape	-	-	-	-	-	-
carrier [ppm]	-	-	-	-	-	-
time [ms]	0.4	0.4	-	0.2	-	-
**Transfer 4**	HC-CP	HC-CP	-	HP-CP	-	-
field [kHz] -^{1}H	60	60	-	60	-	-
field [kHz] -^{13}C	70.7	78.5	-	47	-	-
field [kHz] - ^{15}N	-	-	-	-	-	-
shape	-	-	-	-	-	-
carrier [ppm]	-	-	-	-	-	-
time [ms]	0.9	1.2	-	3.25	-	-
t_1 increments	3072	3072	-	3072	-	-
sweep width (t_1) [kHz]	100	100	-	100	-	-
max. acq time (t_1) [ms]	15.36	15.36	-	15.36	-	-
t_2 increments	1536	136	3072	96	3072	16384
sweep width (t_2) [kHz]	64.127	64.127	100	250	100	100
max. acq time (t_2) [ms]	11.976	11.976	15.36	1.92	15.36	81.9
^{1}H Spinal64 Decoupling power [kHz]	90	90	90	90	90	-
^{1}H Waltz64 Decoupling power [kHz]	-	-	-	-	-	5
interscan delay [s]	2.3	2.3	2.5	2.7	2.7	2.7
number of scans	32	110	20480	2048	640	640

References

1. Kuipers, B. J.; Gruppen, H., Prediction of molar extinction coefficients of proteins and peptides using UV absorption of the constituent amino acids at 214 nm to enable quantitative reverse phase high-performance liquid chromatography-mass spectrometry analysis. *J Agric Food Chem* **2007,** *55* (14), 5445-51.
2. Pradere, U.; Halloy, F.; Hall, J., Chemical synthesis of long RNAs with terminal 5'-phosphate groups. *Chem. Eur. J.* **2017,** *23* (22), 5210-5213.
3. Böckmann, A.; Gardiennet, C.; Verel, R.; Hunkeler, A.; Loquet, A.; Pintacuda, G.; Emsley, L.; Meier, B.; Lesage, A., Characterization of different water pools in solid-state NMR protein samples. *J. Biomol. NMR* **2009,** *45* (3), 319-327.
4. Lange, A.; Luca, S.; Baldus, M., Structural Constraints from Proton-Mediated Rare-Spin Correlation Spectroscopy in Rotating Solids. *J. Am. Chem. Soc.* **2002,** *124* (33), 9704-9705.
5. Wiegand, T.; Schledorn, M.; Malär, A. A.; Cadalbert, R.; Däpp, A.; Terradot, L.; Meier, B. H.; Böckmann, A., Nucleotide binding modes in a motor protein revealed by ^{31}P- and ^{1}H-detected MAS solid-state NMR. *ChemBioChem* **2020,** *21*, 324-330.
6. Shen, Y.; Delaglio, F.; Cornilescu, G.; Bax, A., TALOS+: a hybrid method for predicting protein backbone torsion angles from NMR chemical shifts. *J. Biomol. NMR* **2009,** *44* (4), 213-223.
7. Lewandowski, J. R.; De Paëpe, G.; Griffin, R. G., Proton Assisted Insensitive Nuclei Cross Polarization. *Journal of the American Chemical Society* **2007,** *129* (4), 728-729.
8. Guntert, P.; Mumenthaler, C.; Wuthrich, K., Torsion angle dynamics for NMR structure calculation with the new program DYANA. *J Mol Biol* **1997,** *273* (1), 283-98.
9. Lin, Y. J.; Kirchner, D. K.; Guntert, P., Influence of (1)H chemical shift assignments of the interface residues on structure determinations of homodimeric proteins. *J Magn Reson* **2012,** *222*, 96-104.
